# Supplementary material for: MediYoga compared to physiotherapy treatment as usual for patients with stress-related symptoms in primary care rehabilitation: A randomized controlled trial
Source: PLoS One. 2024 Jun 13;19(6):e0300756. doi: 10.1371/journal.pone.0300756 (PMC11175516; doi:10.1371/journal.pone.0300756)
Supplement: S1 File — (DOCX) [file pone.0300756.s003.docx]

230411

**This is a translation from Swedish to English for the approval of the study**

**from** *EPN Regional Ethics Review Board in Gothenburg.*

***EPN Regional Ethics Review Board in Gothenburg***

Project manager: Dnr: · Exp.2016-08-11

Maria Larsson 658-16

Region Västra Götaland

Community health R&D primary care

Kungsgatan 12, floor 6

411 18 Gothenburg

Head of research: Region Västra Götaland

Present decision-making:

Bo Rolfson, chairman

Bibbi Ringsby-Jansson, assistant scientific secretary (did not participate in case 486-16 due to knowledge of the matter)

**Members with scientific competence**:

Jesper Lundgren (scientific secretary case 486-16)

John Berlin

Elisabeth Björk Brämberg

Karin Klinga Levan

Peter Korp (did not participate in case 486-16 due to knowledge of the matter)

Anna Nordenstam

Ann Svensson

Helle Wijk

**Members representing public interests**:

Bengt Femström

Beatrice Toll

Ingmarie Torstensson

Robert Engstrom

**Project title**: Evaluation of medical yoga, Mediyoga in a group, compared to usual

treatment by a physiotherapist, for people with perceived stress-related unhealth.

**Project ID**: VGFOUGSB-602221

**Minutes of decisions from a meeting with the Regional Ethics Review Board**

**in Gothenburg, Department for Other Research, August 8, 2016**

**Speaker**: Elisabeth Björk Brämberg

*Approved with conditions*

The ethics review board approves the study with the following conditions:

- *that* the group of research subjects is limited to those who are 18 years of age or older, then the questionnaires include sensitive questions and it can be difficult for younger people to take position for participation in a study that aims to test different treatment alternatives for stress.
- *that* the consent form is supplemented in such a way
- *that* the research subjects are given the opportunity to consent to information, where applicable, being retrieved from their records, and
- *that*  there is an opportunity for and information about follow-up within care, in those cases research subjects in the study show signs of

depression or similar.

The decision can be appealed to the Central Ethics Review Board. The letter must be set to

Central Ethics Review Board but sent or submitted to the Regional Ethics Review Board

in Gothenburg.The appeal must be signed by an authorized representative of

the research principal.This person can authorize the researcher to sign. Attach in so

case the power of attorney. The letter must state which decision is being appealed and the change that you requests.The appeal must have been received by the Regional Ethics Review Board no later than three weeks from when the appellant received notice of the decision.

That this transcript in transum conforms to the original certifies:

Barbro Morsing administrative secretary

Diary sheet

EPN in Gothenburg

Project manager: Maria Larsson

Title: Evaluation of medical yoga, Mediyoga in group, compared

with usual treatment by a physiotherapist, for people

with perceived stress-related unhealth.

Project ID VGFOUGSB-602221

Category: A research major

Date Event

2016-07-08 The application was submitted with attachments 2, 4 (AB I), 5, 9, 10, 12

2016-07-11 Appendix 4 (AB II) received

2016-07-13 Fee SEK 5,000 received

2016-08-08 Committee meeting: Accepted with conditions

Print date: 2016-09-30
