# Supplementary material for: MediYoga compared to physiotherapy treatment as usual for patients with stress-related symptoms in primary care rehabilitation: A randomized controlled trial
Source: PLoS One. 2024 Jun 13;19(6):e0300756. doi: 10.1371/journal.pone.0300756 (PMC11175516; doi:10.1371/journal.pone.0300756)
Supplement: S2 File — (PDF) [file pone.0300756.s004.pdf]

Start-up Package 1

from 40 min

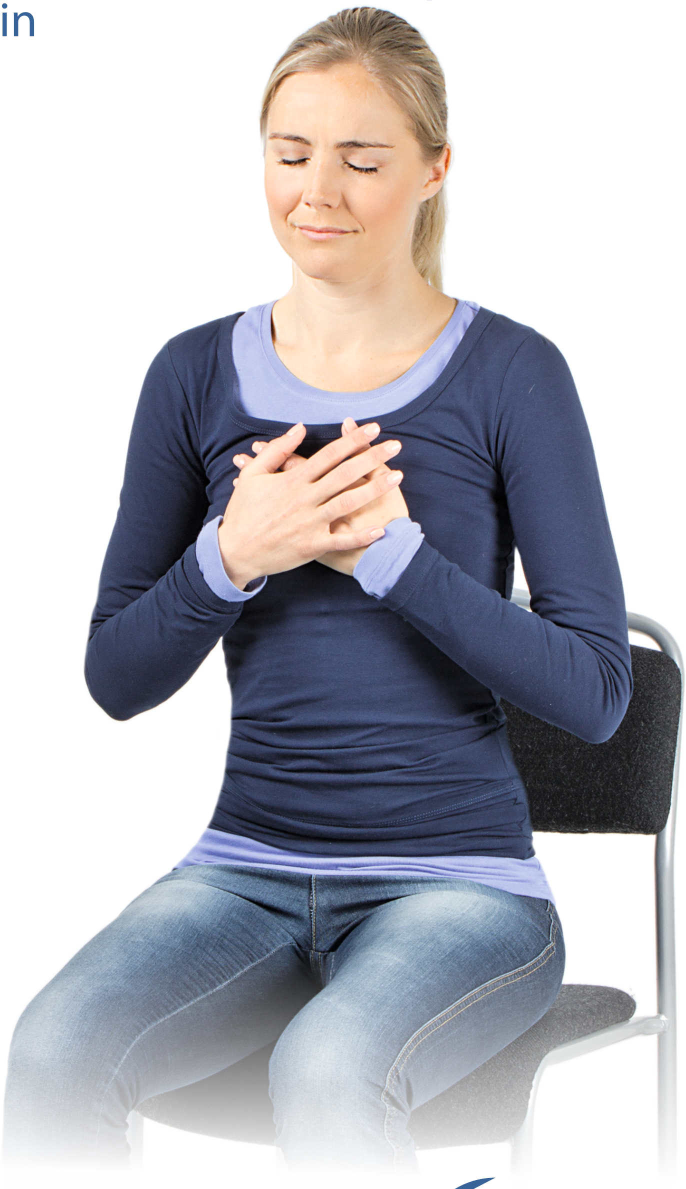

MEDIYOGA®

Research - Knowledge - Education

Contents

MediYoga - Start-up package 1

- What is MediYoga?
- To think about before you start
- About yoga
- About meditation
- Breathing techniques - Long, deep breathing
- Yoga exercise, Spinal flex
- Yoga exercise, Sat Kriya
- Meditation, For the heart - Guru Ram Das
- Meditation - Kirtan Kriya
- MediYoga and research

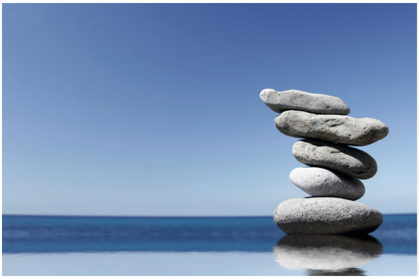
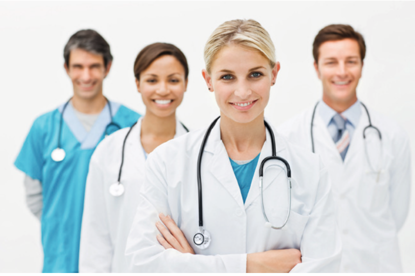

Disclaimer.
The programs described on this CD is not intended to be a substitute for medical care and advice. You are advised to consult with your health care professional with regards to matters relating to your health. Yoga can be challenging and hard work but it should never cause pain.

What is MediYoga?

MediYoga is a therapeutic form of yoga

Each year, we train hundreds of instructors and yoga therapists in Sweden and Norway. What makes us unique and differentiates us from all other forms of yoga is that everyone who works with MediYoga, instructors, teachers and yoga therapists, have health profession qualifications and understand the effects of yoga from a medical perspective.

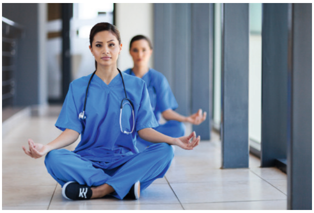

MediYoga - was established and developed by Göran Boll at IMY, the Institute for Medical Yoga, in Stockholm, Sweden. MediYoga has its origins in classic Kundalini yoga and started to take shape as early as 1998, when an initial partnership project was launched with the Karolinska Institute in Stockholm. This consisted of a study of what yoga could offer for patients with chronic back pain. Many different studies have been undertaken since then involving MediYoga and its effects on various patient groups and medical disorders in general. These also include studies performed at large companies such as the Post Giro Stress project in 1999 and the Swedish Enforcement Authority Stress project in 2009. Since 2004, MediYoga has been one of the most popular rehabilitation options for employees on long-term sick leave at AstraZeneca.

Our courses are primarily aimed at health care professionals where nurses, physiotherapists, therapists and doctors have learned about long, deep breathing and other simple techniques they can teach their patients and clients. You will find many of them in our register of instructors, ”Find a MediYoga Instructor near you” at our website.

Our vision is one of yoga as a natural part of society and an everyday feature of people's lives. This is where it is needed. And this is why we are regularly involved in new research projects, the development of courses plus products and services to make yoga readily accessible and available where it benefits people – right in the heart of hectic everyday life.

Our course programme includes:

- **Instructor in MediYoga courses, 1 term**
- **Teacher & Therapist in MediYoga courses, 3 terms**
- **Courses in MediYoga for Children and Young People, 4 days**
- **Workshops for people who wish to learn how MediYoga can help with e.g. cancer, burn out etc.**

For more information on our courses go to www.mediyoga.com

Introduction

Our world is becoming an increasingly stressful place and we need several powerful tools to create a better balance in our lives and our everyday existence.

MediYoga is a very powerful tool in combating stress, burn out and other imbalances and it is simple to use. There are yoga exercises to suit everyone, yogic techniques that you can easily incorporate into your everyday life. Yoga has been a source of inspiration for many modern disciplines and treatment methods.

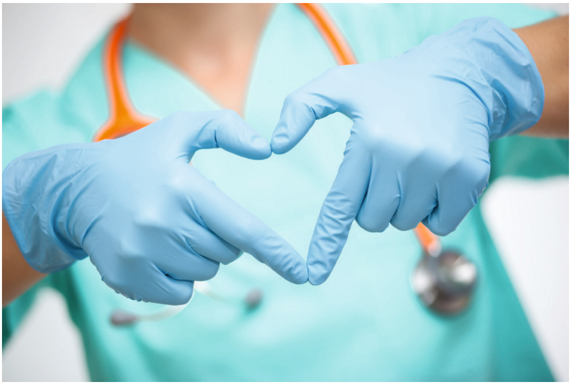

To think about before you start

- **Organise a space where you can be undisturbed.**
- **Practise Yoga barefoot if possible.**
- **Follow the sequence of exercises in a yoga programme.**
- **Do not exceed the times shown, follow the instructions and do the exercises to the best of your ability.**
- **Do not eat just before doing a yoga or meditation programme. The digestion process makes training more difficult.**
- **Drink water after finishing a yoga programme or meditation.**
- **Take it easy immediately after doing yoga exercises and meditation. Take things gently when you go back to your everyday tasks in order to retain the feeling of balance and tranquillity for as long as possible after finishing the programme.**

What does it mean?

Tuning in

All MediYoga programmes start by tuning in. Tuning in is a bit like blowing a whistle to start a football match, the signal that it is time to begin. This is what you do: Close your eyes. Sit with the palms of your hands together in front of your chest, thumbs towards your breastbone. Tune in with the mantra ONG NAMO GURU DEV NAMO three times. The mantra means you are opening yourself to your inner strength and your inner wisdom.

Focus: Sat Nam and the third eye

A good way of keeping your concentration is to keep your eyes closed and focus on a point midway between your eyebrows, the third eye. Maintain this focus both during and between exercises. According to yogic tradition, the third eye is the seat of intuition and knowledge about yourself. Always take long, deep breaths through the nose unless the exercise specifically requires some other form of breathing. Link the mantra SAT NAM to your breathing, think SAT when breathing in and NAM when breathing out. This helps you keep the focus on the here and now, to make you aware of how the mind and body react during the exercises.

Neck lock

In a sitting position you should aim to pull in your chin slightly so your neck is in correctly aligned with your back. This facilitates the flow of energy along the spine all the way to the brain.

Root lock

A muscle contraction at the base of the spine that generally concludes every yoga exercise. Breathe out and empty your lungs. Hold your breath out. Contract and hold the muscles around your anus and around the sex organ (like stopping the flow of urine). Finally, contract the muscles of the lower abdomen and the navel point toward the spine. Hold all three for 5-15 seconds. Breathe in, release the root lock, breathe out and relax. The root lock frees Kundalini energy in the body and balances the pelvis.

Breath of fire

A powerful breathing technique that boosts circulation and energy level in the body. Take rapid breaths in and out through the nose, 1-3 times per second without pausing between inhalation and exhalation. Relax your chest and use your stomach to assist your breathing. When you breathe in, push your tummy out and when you breathe out, pull your tummy back in again. As though you were panting through the nose; rapidly and lightly at the top of your chest. People often feel a bit dizzy when first doing breath of fire, but provided the stomach is controlling your breathing in the right way you will never suffer hyperventilation. What is important here is to make sure you take the same deep breaths in and out.
No breath of fire if you are menstruating or pregnant.

What does it mean?

Tuning out

At the end of the yoga programme and/or meditation you tune out with SAT NAM three times. SAT NAM means - I am true.

Important!

If you are female: Take it easy with yoga when menstruating. Meditate instead. Certain exercises such as e.g. breath of fire and root lock should be skipped during your period. If you are pregnant, speak to a yoga teacher first so you know what exercises you can do. Applicable to everyone: No yoga in association with the consumption of alcohol or other drugs.

Keep an eye on how the exercises are affecting you. Something else that is an important part of your yoga training is to listen to your body. If an exercise gives you physical pain, do it more slowly, for a shorter time – or not at all. Yoga should be fun – and never painful.

About yoga

Yoga is a large concept that spans many different aspects of life and everyday living. The word yoga is often said to mean balance. It comes from Sanskrit and its root is the word Yuj that literally means to yoke or unite. Yoga literally aims to assume the yoke, to exert and discipline yourself, to balance your mind and body, body and soul. Yoga is a system of physical, mental and spiritual training. A common misconception is that yoga is a system of physical exercises and postures. The fact is, yoga is a complete science for life, a philosophy of life where physical postures (asanas) are just one of many components.

The practice of yoga goes back a very long time. Archaeological excavations in India and Pakistan date yoga to at least 4,000-5,000 years ago. And many yoga masters argue that yoga is far older than that. For a long time, yoga was the preserve of a very small number of people, but today, these techniques are practised by hundreds of millions of people the world over. For millennia, yoga has been wrongly associated with various theological and philosophical systems. Yoga is not in itself a religion and nor is it linked to any specific religion. Yoga is first and foremost a practical, physical, mental and spiritual discipline that leads to spiritual enlightenment through personal exercise. Yoga is simply a tool that enables you to delve into your own human nature.

Yoga does not claim to be the only or the true path to salvation. No one is ever excluded from practising yoga. Quite the reverse - yoga exercises can be – and are- performed by all sorts of people all round the world, regardless of religious persuasion and ethnicity. When you practise yoga, you do not need to believe in anything other than the possibility of being able, through your yoga training, to transform and reorient yourself towards reaching your full potential and come into contact with your innermost self – the person you really are.

About yoga

Yoga exercises are performed slowly and in a controlled manner in order to develop physical suppleness, create mental relaxation and lead to enlightenment. Breathing plays an important and fundamental role in yoga. According to yogic philosophy, breathing embodies the vitality of life, or Prana. For most people, breathing is an unconscious function. It can, however, be consciously changed by using various breathing techniques, which in their turn also affect our well-being. Breathing is also a direct reflection of different emotional states. If we are under stress, our breathing is rapid and shallow. If we are relaxed, we breathe deeper, calmer breaths. Which means by consciously changing the way we breathe we can directly affect the way we feel and create physical, mental and emotional harmony inside ourselves. Breathing is a unique way of being able to directly affect the subconscious processes in our mind and body.

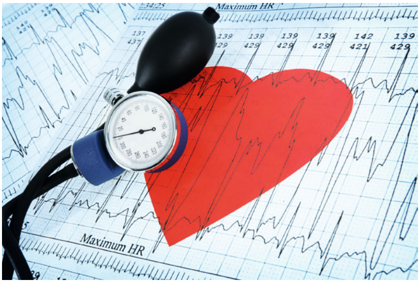

For example, yoga affects:

- Breathing and lungs
- Musculature
- Digestive system
- Glandular system
- Lymphatic system
- Nerve system and brain

About meditation

Meditation is an important part of MediYoga. The yoga programme is normally concluded with meditation and there are many ways to meditate.

It reduces the body's negative reaction to stress. Measurements of muscle tension during meditation have shown this goes down to virtually zero. This means meditation can alleviate high blood pressure, headaches and chronic pain conditions. Blood circulation is improved and EEG measurements show that brain activity during meditation is similar to sleep which can be of help to people with sleep disorders. As with yoga, meditation provides breathing space, space to breathe. Meditation aims to strengthen the will and your ability to organise your life in the best way.

About meditation

A few more words on meditation:

- **Meditation increases concentration and mental alertness**
- **Meditation helps you live with greater awareness and balance inside yourself.**
- **Meditation gives you an inner calm that helps you develop your intuition**
- **Meditation makes you less reactive, i.e. less governed by your feelings alone**

Meditation requires concentration, endurance and time, e.g. 11 minutes twice a day is recommended. Most people can learn to meditate. To a certain extent it is about “tidying the attic” – not always fun but much needed and satisfying when you have done it.

When you start to meditate you can notice how your thoughts and mind take you with them. You may get a sense of losing concentration but this is a totally normal reaction. To regain focus, use mantra, breathing or the technique you use in meditation. Note how you can get thoughts that relate to current problems. Deep-seated stress can appear in the form of superficial thoughts.

There are numerous different ways of meditating and it is basically about different paths to the same goal. E.g. concentration, breathing, aural, mantra, visualisation and movement meditations. MediYoga contains hundreds of different meditations for relaxation, balance, depression, energy, heart, healing, brain, intuition, concentration, creativity, mental control, nerve strength, self-esteem, stress, willpower and vitality - to name just a few.

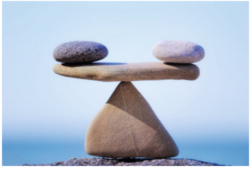

- Shed all expectations when you meditate
- Behold
- Feel it
- In the very depth of you, you know who you are and the direction you are going in

Long, deep breathing

Long, deep, conscious breathing is fundamental to MediYoga.

The long, deep, breathing consist of three parts:
Stomach breathing. Place your hands on the lower part of your stomach. Breathe in through the nose and guide the breath down into your belly and stomach. Feel your hands being lifted towards the ceiling.Feel that your breathing is also filling the curve of your back and along your spine, hold your breath in for a moment, feel your stomach. When it is time to breathe out, slowly relax your stomach and breathe out all the way.

Rib cage breathing. Place your hands on your ribs, relax your shoulders. Breathe in through the nose and fill your rib cage, along the sides first and then along the spine and forward in the chest. Let your rib cage expand. Breathe out through the nose and let your rib cage move down and in. Feel the movement under your hands. Let your rib cage expand more and more for each breath.

Collarbone breathing. Place one hand so your thumb and index finger touch your collarbone. Breathe in deeply through the nose, lift your collarbone in a straight line such that your entire rib cage is raised without your back and shoulders doing the same. Hold your breath for a few moments, then breathe out through the nose and lower your collarbone and rib cage. Feel the movement under your fingers.

These three parts have been combined together into long, deep, yogic breathing.
The breathing should be like a wave movement where the stomach rises, then the ribcage; the ribcage sinks then the stomach. After breathing out, with empty lungs, see if you can rest for a few seconds; let the body take the initiative for the next in breath. A good way of training your breathing when first starting is to lie on your back. Hold one hand on your stomach and one on your chest and feel the movement of your breathing. Unless some other kind of breathing technique is specified for an exercise, you should always take long, deep breaths, even between exercises.

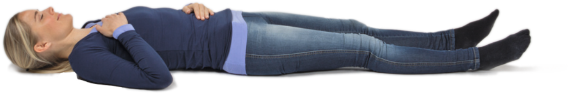

Long, deep breaths:

- **Create calm and relaxation, provide clarity and patience**
- **Reduce toxins and mucus from your lungs and airways**
- **Help blood circulation**
- **Prevent the accumulation of cholesterol in the blood**
- **Stimulate chemical balance in your brain and regulate pH**
- **Increase the flow of spinal fluid to the brain**
- **Mitigate depression, uncertainty and fear**
- **Free up blockages in the pranic flow**
- **Strengthen your electromagnetic field, your aura**
- **Affect the pituitary gland, open up your intuition etc.**

Spinal flex

This exercise is one of the most fundamental in MediYoga. It flexes the spinal column, softens and increases blood flow through the musculature of the spine, it stretches the respiratory muscles in the chest, stimulates flow in the lymphatic vessels along the spine and the flow of spinal fluid around the spinal cord. From the yogic perspective it opens the flow of energy in the large spinal channel, Sushumna, the main meridian in the body where all the main chakras sit.

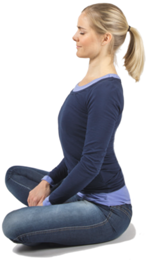

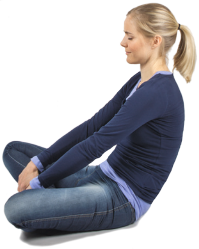

3 minutes

Sit with your legs crossed. Back straight. Place your hands on your ankles.Close your eyes. Start by tilting your pelvis forwards, pressing your lower back forwards and lifting your chest forwards. To flex backwards, tilt the pelvis back, allow your lower and upper back to round, shoulders coming slightly forwards.

Your head should stay neutral on your spine, neither looking up nor down, your nose is always pointing to the front. As you flex forward breathing in and breathing out when you flex back. Think the mantra SAT when you breathe in and NAM when you breathe out.

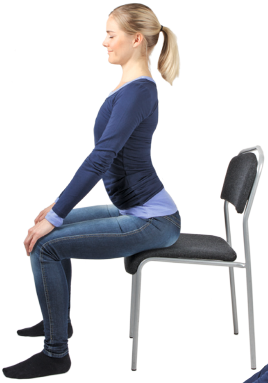

You can do this exercise just as well sitting on a chair or on the edge of the bed. Sit with your back straight slightly to the front of the seat, away from any back rest. Knees aligned with your hips or slightly further apart, hands on knees.

Breathe in and flex your spine and chest forwards. Breathe out flex your spine backwards. Your head should stay neutral on your spine, neither looking up nor down, your nose is always pointing forwards Do the Spinal flex for 3-5 minutes, ideally several times a day.

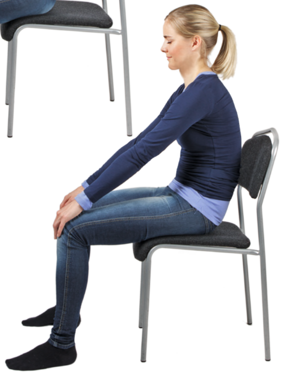

Sat Kriya

3-11 minutes

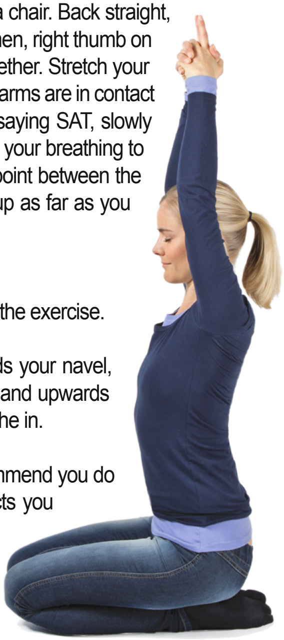

Kneel on the floor sitting on your heels alternatively sitting on a chair. Back straight, chin tucked in. Interlace your fingers, left thumb on top for women, right thumb on top for men. Straighten your index fingers and press them together. Stretch your arms up towards the ceiling, elbows straight so that your upper arms are in contact with your ears. Do a powerful root lock at the same time as saying SAT, slowly and consciously. Release the root lock as you say NAM. Allow your breathing to take care of itself, eyes closed focusing on the third eye, the point between the eyebrows. Conclude the exercise by breathing in, stretching up as far as you can, breathe out, hold your breath out and do a root lock.

Important:
Rest afterwards for at least the same length of time as you did the exercise.

Root lock: Engage the pelvic floor, drawing it upwards towards your navel, at the same time draw the lower part of the stomach inwards and upwards towards the spine.. Hold for about 10 sec, then relax and breathe in.

Sat Kriya is one of the basic exercises in MediYoga. We recommend you do this exercise at least 3 minutes every day. It profoundly affects you and has a powerful balancing effect on the three lowermost chakras. In addition to the root chakra, it strengthens the whole of your sexual system in the body, releases phobias and stimulates a free, natural energy flow through your entire system. It stimulates creativity and the body's capacity for self-healing, and builds self-esteem. All the internal organs in the belly are given a rhythmic massage during the exercise. The heart is boosted and stimulated. Energy flow is stimulated in the body. SAT Kriya is classed as a complete yoga session in its own right when done for at least 11 minutes. Start by doing it for 3 minutes and then slowly work up to 11 mins. Allow it to take time.

Respect the power in the technique and always rest afterwards - for at least the same length of time you spend on the exercise.

When menstruating or pregnant:
Sit on your heels with your hands on your knees. Left hand cupped in the right hand, palms facing upwards. Relax your arms, shoulders and jaw. Whisper the mantra SAT NAM in time with your breathing, breathe in while whispering SAT and breathe out while whispering NAM, no root locks.

If you live a stressful life and feel you do not have much time for yoga or other techniques – practice Sat Kriya, 3-5 minutes a day. It works on your, mind, body, consciousness and sub consciousness. It creates balance, gives you energy and deepens your insight.

Heart meditation

11 minutes

Sit with your legs crossed and your back straight, alternatively siting on a chair slightly away from the backrest. Place the palm of your left hand on the middle of your chest, and right hand over your left hand. Relax your shoulders, elbows and jaw. Close your eyes, focus on your heart and listen to or chant the mantra:

GURU GURU WAHE GURU - GURU RAM DAS GURU

The meaning of which translates approximately as:
Wisdom and insight will come to you - As a servant of the eternal.

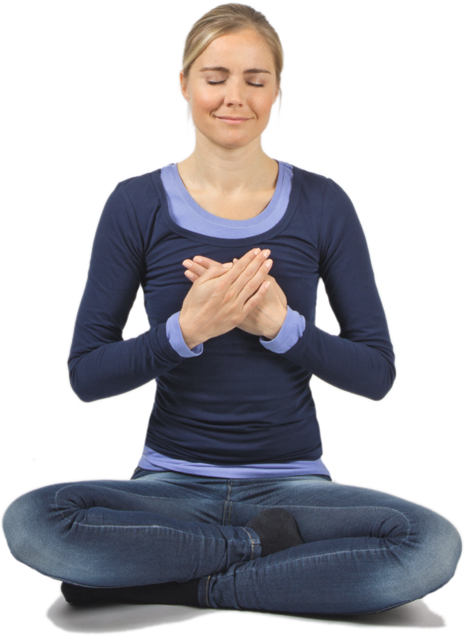

This is a powerful mantra for healing and protection. If it is chanted in association with a difficult situation, its vibrations are said to be able to clear up things that are unclear in this situation, provide protection and heal that which needs healing. The mantra creates an inner meditative peace. The impossible suddenly becomes easy and very possible. The first line of the mantra is said to transport your mind to the innermost source of knowledge. The second line is said to be able to take us to a higher consciousness.

Kirtan Kriya

12-31 minutes

Western research into this meditation shows that it improves the mind. According to the yogic approach it creates complete mental balance in the mind and changes the polarity in the body's electrical field in a way that strengthens and balances the electromagnetic field – the aura – around your body. It is also said to provide a healing effect on emotional wounds. It flushes away mental and emotional blocks. It is said to be particularly powerful for women as it prevents the psyche from being attracted to negative relationships.

Sit in your meditation posture cross legged on a cushion on the floor alternatively sitting on a chair. Back straight and chin slightly tucked in. Close your eyes and focus on the third eye, the point between your eyebrows. Your hands resting gently on your knees or thighs. Repeat the mantra as instructed below for 12 or 31 minutes:

SA – TA – NA – MA

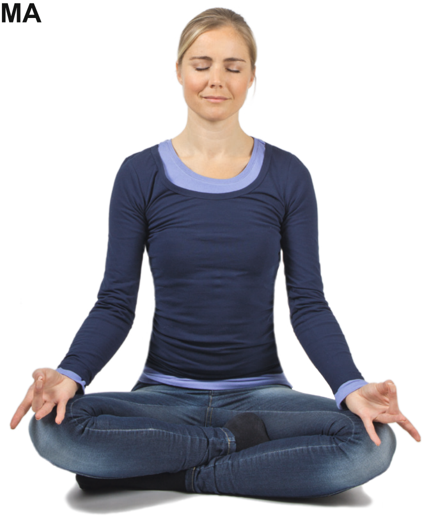

- **On SA press your thumb and tip of your index finger together. The index finger (Jupiter finger) represents Wisdom, Knowledge and Excess.**
- **On TA press your thumb and tip of your middle finger together. The middle finger (Saturn finger) represents Focus, Discipline, Structure and Boundary setting**
- **On NA press your thumb and tip of your ring finger together. The ring finger (Sun finger) represents Life and Relationships.**
- **On MA press your thumb and tip of your little finger together. The little finger (Mercury finger) represents communication.**
- **The thumb represents Consciousness and Ego.**

Kirtan Kriya

12 minutes (31min)

2 min, (5 min) chant/sing the mantra Sa Ta Na Ma aloud
2 min, (5 min) whisper the mantra
4 min, (10 min) think the mantra silently to yourself, continue the finger movements.
2 min, (5 min) whisper the mantra
2 min, (5 min) chant/sing the mantra aloud

To end:
Breathe in and stretch your arms up in the air, breathe in and form a claw with your fingers and shake your hands vigorously above your head for a few moments. Bring your arms back down again and sit in quiet contemplation for a little while before going back to everyday activities again.

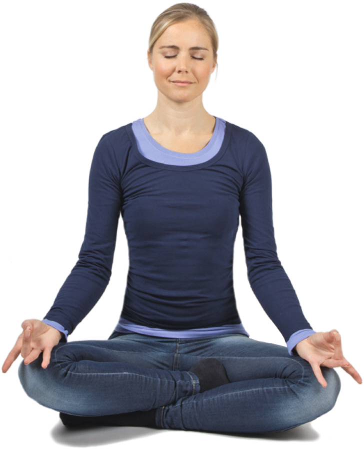

SA - Eternity, cosmos or beginning
TA - Life or existence
NA - Death
MA - Rebirth

SA TA NA MA is the core form of the mantra SAT NAM, I am truth, which is the most common mantra in MediYoga. SA TA NA MA stands for the cycle of creation, the eternal cycle of rebirth. Life and individual existence comes from eternity. Out of life comes death and change. Out of death comes the rebirth of consciousness in eternal joy and out of eternal empathy life starts again once more. According to yoga, consciousness has three different voices:

Normal tone of voice - The worldly language of humans.
Whispering – The language of loving, longing and feeling.
Silent, mental – The spiritual and eternal language

Advice: One way of avoiding a headache when you do this meditation is to visualise light/energy entering the top of your head and then exiting through the third eye in your forehead. The light forms an L shape and emerges through the forehead. In SA you think S at the top of your head and A in the third eye. According to yoga, energy moves along a golden ribbon that links the pineal gland with the pituitary gland.

Advice: If your mind begins to wander when you start thinking the mantra silently inside yourself, go back to whispering for a moment or two, then back to aloud, down to a whisper and then back into silence again. If you need to do this to focus your mind, you can repeat this several times during the silent phase.

Research – Knowledge – Education

Examples of studies involving MediYoga

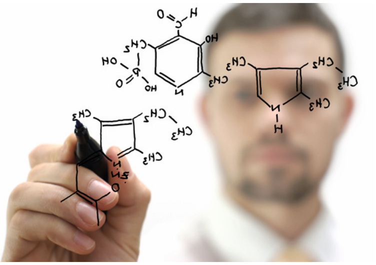

Atrial fibrillation and MediYoga, 2012
Danderyd Hospital, Stockholm. Report completed, awaiting publication.

Strokes and MediYoga, 2012-2014
Danderyd Hospital, Stockholm.

ME / Chronic Fatigue Syndrome and MediYoga, 2011-2012
Danderyd Hospital, Stockholm.

Chronic back problems/pain and MediYoga, 1998, updated in 2011.
Karolinska Hospital, Stockholm

Heart failure and MediYoga, 2011-2012
Huddinge Hospital, Stockholm. Report completed, awaiting publication.

Alcohol problems and MediYoga, 2012-2013
Karolinska Institute, Karolinska Hospital, Stockholm.

First Aid and MediYoga, 2011-2012
Nora Clinic/Primary Care. MediYoga is offered to patients as first aid for stress related illnesses such as burn out, nervous problems, anxiety, depression and sleeping disorders. Report completed, awaiting publication.

Leukaemia and MediYoga, 2011-2012
Halmstad University, Children aged 8-12 years

High blood pressure and MediYoga, 2010-2011
Svedala Clinic/Primary Care. Report completed, awaiting publication.

Heart attacks and MediYoga, 2009
Danderyd Hospital, Stockholm. Abstract, no report published.

Atrial Fibrillation and MediYoga, 2013-2017
Danderyd Hospital, Stockholm. Major new study involving several hundred patients. Starting in spring 2013

MediYoga's own studies
Since 2010, IMY (Institutet för Medicinsk Yoga) have compiled the responses from numerous "before and after" questionnaires completed by students/clients that MediYoga instructors and therapists send in.

About this production

Produced by **Göran Boll** and **Elisabeth Engqvist** for **Mediyoga Internationa**l.

www.mediyoga.com

Narrator: Kim Westmoquette
Scriptwriters: Göran Boll and Elisabeth Engqvist
Model: Madeleine Wilhelmsson
Photo: David Boll
Layout: Kristoffer Folin, Fredrik Wännman

Music:
Sat Nam (Music: Fredrik Wännman, trad lyrics)
Sa Ta Na Ma (with Matilda Thompson, Music: Fredrik Wännman, trad lyrics)
Waves of Aum (Music: Fredrik Wännman)
Guru Ram Das (Music: Ann Britt Ljusberg IMY Publishing, trad lyrics)

Speech and music recorded at Ramtitam Studios www.ramtitam.se. Music performed by Fredrik Wännman except Guru Ram Das, performed by Ann- Britt Ljusberg. Birdsong recorded by Patrik Åberg. All music is licensed to MediYoga International.

Product content and design is a joint production between
Mediyoga Sweden, **Mediyoga International** and **Ramtitam.**

Executive Producer and Publisher:
Elisabeth Engqvist for **Mediyoga International.**

All rights reserved℗&©2012 MediYoga® Sweden AB
and MediYoga International.

Why MediYoga?

Scientific research is increasingly showing that stress in its various forms is the underlying cause of most of what we call illnesses and that powerful tools are needed to re-establish and maintain balance in our lives.

In collaboration with several leading hospitals in Sweden, research on Mediyoga has been carried out since 1998 that demonstrate yoga as a practical tool that really works. These studies have shown measurable effects on back and sleep problems, high blood pressure, ventricular fibrillation, various emotional problems and other disorders. Mediyoga is now recommended by healthcare professionals throughout Sweden and Norway. This is a simple and therapeutic form of yoga that anyone can do, whatever their physical or psychological limitations.

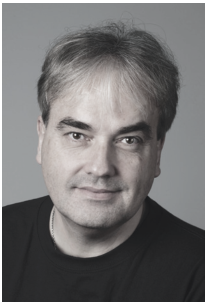

These simple MediYoga exercises can be done anywhere and at any time of the day or night. We hope this programme of breathing, gentle physical exercises and meditation can help you in the direction of better health and balance.

Welcome to the fantastic world of yoga!

Göran Boll
Founder of MediYoga

On the CD

MediYoga Start-up Package 1

1. Introduction 3.50
2. Tuning in 2.22
3. Long deep breathing 10.49
4. Spinal flex 3.58
5. Sat Kriya when menstruating 1.09
6. Sat Kriya, 3 minutes 5.15
7. Sat Kriya, 11 minutes 12.52
8. Relaxation 6.40
9. Heart Meditation - Guru Ram Das 11.59
10. Meditation Kirtan Kriya - Sa Ta Na Ma 12.57
11. Tuning out 1.49

SUP-1 BL v 1.4

Music

The music on this CD can be purchased separately.

Sat Nam and Waves of Aum on the CD:
MediYoga Music and Mantras - Ramtitam One

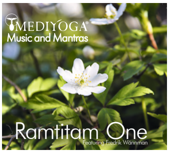

Sa Ta Na Ma on the CD:
MediYoga Music and Mantras - Ramtitam Two

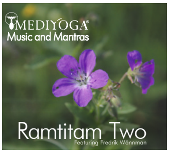

Guru Ram Das on the CD:
MediYoga Music and Mantras - Harmony One

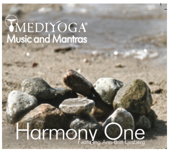

MEDIYOGA®

The power and the tools to change your
future direction are there within you
