# Supplementary material for: MediYoga compared to physiotherapy treatment as usual for patients with stress-related symptoms in primary care rehabilitation: A randomized controlled trial
Source: PLoS One. 2024 Jun 13;19(6):e0300756. doi: 10.1371/journal.pone.0300756 (PMC11175516; doi:10.1371/journal.pone.0300756)
Supplement: S3 File — (PDF) [file pone.0300756.s005.pdf]

Startpaket 2

från ca 50 min

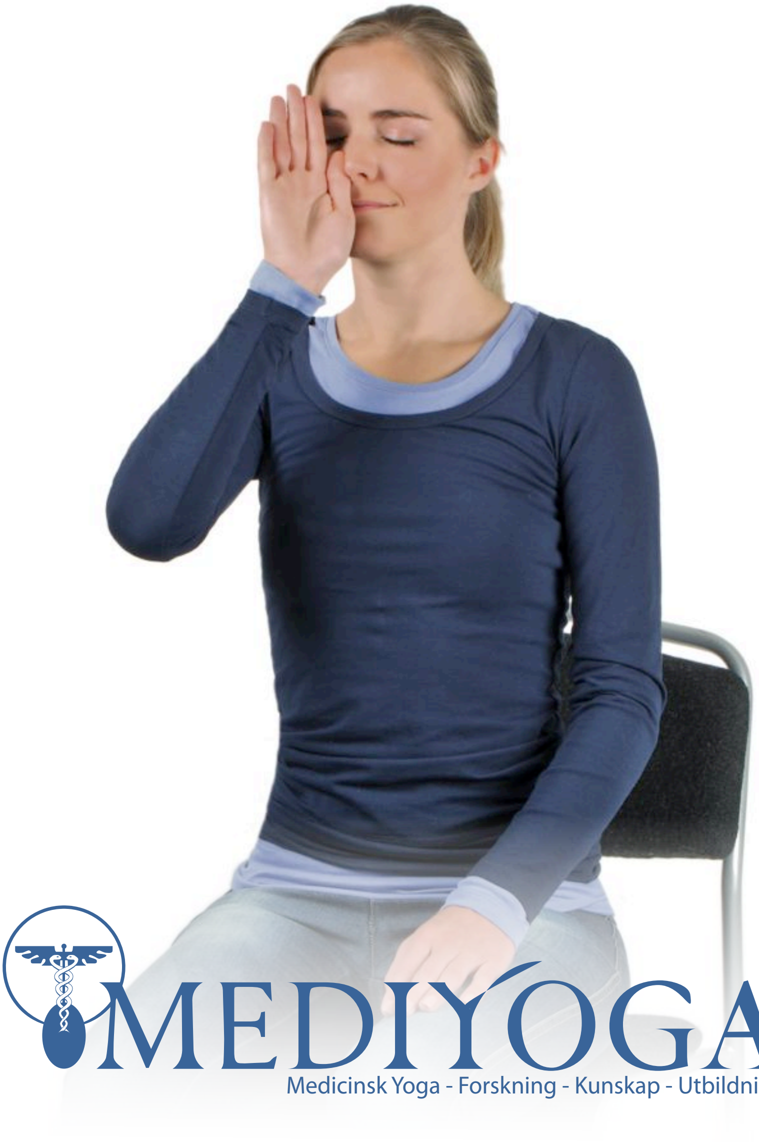

MEDIYOGA®

Medicinsk Yoga - Forskning - Kunskap - Utbildning

Innehållsförteckning

MediYoga - Startpaket 2

Innehåll häfte:

- Vad är MediYoga?
- Att tänka på innan du börjar
- Om yoga och meditation
- Andningsteknik - Långa, djupa andetag på rygg
- Yogaövning - Suficirklar
- Yogaövning - Ryggböjningar
- Yogaövning - För skuldror och nacke
- Meditation - Grundläggande andningsmeditation
- Meditation - Starka nerver, Lugnt sinne
- MediYoga och forskning

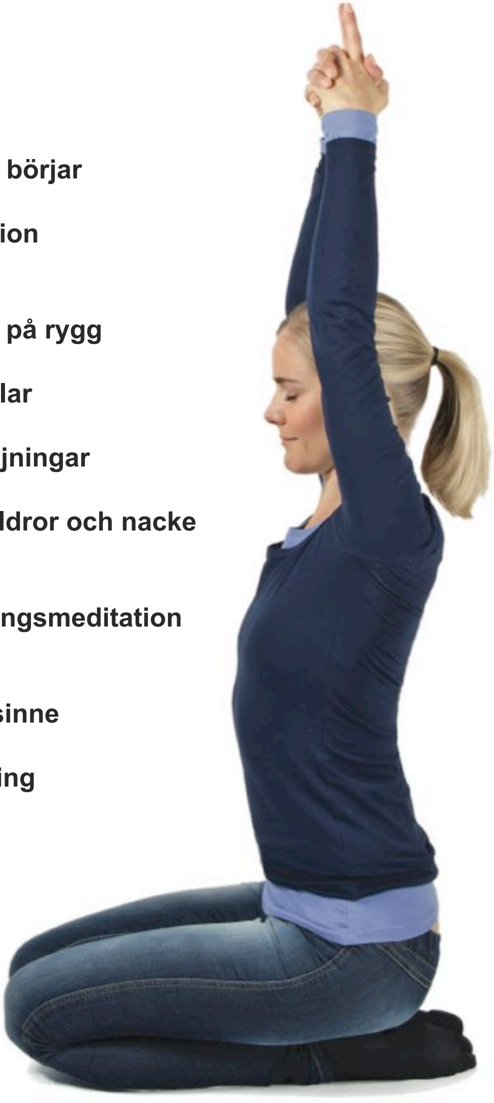

Om MediYoga - Medicinsk Yoga

MediYoga är en terapeutisk yogaform

Varje år utbildas hundratals sjuksköterskor, sjukgymnaster, terapeuter och läkare i MediYoga. De lär sig långa, djupa andetag och andra enkla tekniker som de i sin tur lär ut till sina patienter och klienter. Alla vi som instruerar Mediyoga har medicinsk kompetens och förstår yogans effekter ur ett medicinskt perspektiv. Du hittar många av våra instruktörer och yogaterapeuter i vårt instruktörsregister ”Vem kan lära mig” på vår hemsida.

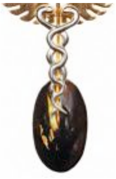

MediYoga – medicinsk yoga, är grundat och framtaget av Göran Boll på IMY, Institutet för Medicinsk Yoga, i Stockholm. MediYoga har sitt ursprung i klassisk Kundaliniyoga och började ta form redan 1998, då det första samarbetet med Karolinska Institutet startade. Då gällde det en studie om vad yoga kan göra för patienter med kroniska ryggsmärtor. Sedan dess har det genomförts många olika studier på MediYoga och dess effekter på olika patientgrupper och ohälsa i stort. Det har även gjorts flera studier på stora företag som Post Girots Stressprojekt 1999 och Kronofogdens Stressprojekt 2009. MediYoga är sedan 2004 ett av de mest etablerade rehabiliteringsalternativen för långtidssjukskrivna på Astra Zeneca.

Vi arbetar för att yoga ska finnas som en naturlig del i samhället, i människors vardag, i skolan, på arbetet, i sjukvården etc. Det är där den behövs. Vi gör det genom nya forskningsprojekt, utveckling av utbildningar samt produkter och tjänster för att yogan ska bli lättillgänglig och finnas där den gör nytta – mitt i den snabbt snurrande vardagen.

Vi har bland annat:

- **Utbildningar till Instruktör i MediYoga, 1 termin**
- **Utbildningar till Lärare och Terapeut i MediYoga, 3 terminer**
- **Utbildningar i MediYoga för Barn och Ungdomar, 4 dagar**
- **Workshops för dig som vill veta vad MediYoga kan göra vid t.ex Stress eller Migrän**

Titta in på hemsidan för mer information om våra utbildningar www.mediyoga.se

Inledning

I den allt mer stressfyllda värld vi lever i behövs idag fler kraftfulla verktyg för att bättre skapa balans i våra liv och vår dagliga tillvaro.

MediYoga är ett mycket kraftfullt redskap mot stress, utbrändhet och andra obalanser och det är enkelt att använda. Det finns yogaövningar och yogiska tekniker som passar för alla, som du enkelt kan använda i din vardag. Många moderna discipliner och behandlingsmetoder har hämtat sin inspiration just från yogan.

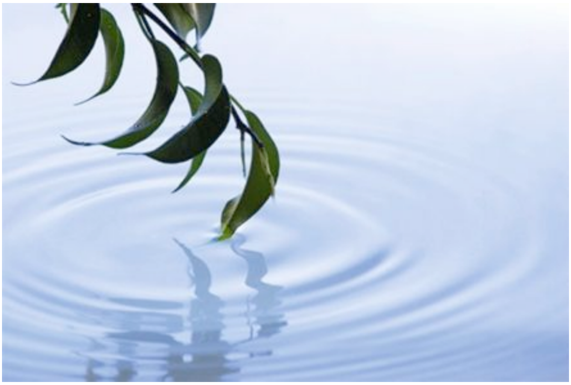

Att tänka på innan du börjar

- **Gör i ordning en plats åt dig där du kan vara ostörd.**
- **Träna gärna barfota.**
- **Håll ordningsföljden mellan övningarna i ett yogaprogram.**
- **Överskrid inte tidsangivelserna, följ instruktionerna och träna efter bästa förmåga.**
- **Ät inte för nära inpå ett yoga- eller meditationsprogram. Matsmältnings-processen försvårar träningen.**
- **Drick vatten efter genomfört yogaprogram eller meditation.**
- **Ta det lugnt direkt efter yogaövningarna och meditationen. Sträva efter att mjukstarta in i vardagen igen för att bära med dig känslan av balans och stillhet långt efter det att programmet är slut.**

Vad betyder det

Intoning

MediYoga börjar alla yogaprogram med en så kallad intoning. Intoningen kan jämföras med en startsignal på en fotbollsmatch, att nu är det dags att börja. Så här gör du: Slut ögonen. Sitt med handflatorna samman framför bröstet, tummarna mot bröstbenet. Tona in dig med mantrat ONG NAMO GURU DEV NAMO tre gånger. Mantrat betyder att du öppnar dig för din inre styrka och din inre visdom.

Fokus: Sat Nam och tredje ögat

Ett bra sätt att hålla koncentrationen är att ha ögonen slutna och fokus på en punkt mitt mellan ögonbrynen, tredje ögat. Håll fokus både under och mellan övningarna. Enligt yogisk tradition är tredje ögat sätet för intuitionen, kunskapen om dig själv. Andas alltid långa, djupa andetag genom näsan om inte en övning specifikt kräver en annan form av andning. Koppla mantrat SAT NAM till andetaget, tänk SAT på in- och NAM på utandning. Det hjälper dig att hålla fokus här och nu, för att göra dig själv uppmärksam på hur kropp och sinne reagerar under övningarna.

Nacklås

I sittande ställning strävar du efter att dra in hakan en aning så nacken kommer i rät linje med ryggen. Det underlättar energiflödet längs ryggraden hela vägen upp till hjärnan.

Rotlås

En muskelkontraktion i nedre delen av bålen som oftast avslutar varje yogaövning. Andas ut all luft. Håll andan ute. Knip ihop anusmuskeln och håll den. Dra muskulärt upp urinvägs-trakten och håll även den samt dra naveln in mot ryggraden. Håll alla tre under 5-15 sekunder. Andas in, släpp rotlåset, andas ut och slappna av. Rotlåset frigör Kundalinienergi i kroppen och balanserar bäckenet.

Eldandning

En kraftfull andningsteknik som sätter fart på cirkulationen och ökar energinivån i kroppen. Du andas snabbt in och ut genom näsan, 1-3 ggr/sekund utan paus mellan in- och utandning. Slappna av i bröstkorgen och tag magen till hjälp i andetaget. När du andas in putar magen ut och när du andas ut dras magen tillbaka in igen. Precis som att flämta genom näsan; snabbt och lätt högt uppe i bröstet.

Det är lätt att bli yr av eldandning i början, men så länge magen på rätt sätt styr andetaget blir det aldrig fråga om hyperventilation. Viktigt här är att in- och utandning alltid ska vara lika långa. Ingen eldandning vid menstruation eller graviditet.

Vad betyder det?

Uttoning

Efter avslutat yogaprogram och/eller meditation tonar du ut dig med SAT NAM tre gånger. SAT NAM betyder - Jag är sann.

Viktigt!
Om du är kvinna: Ta det lugnt med yoga under menstruation. Meditera hellre. Vissa övningar, t.ex. eldandning och rotlås hoppar du helt över dessa dagar. Om du är gravid, tala med en yogalärare innan så du vet hur du kan träna. Gäller alla: Ingen yoga i samband med förtäring av alkohol eller andra droger.

Var uppmärksam på hur övningarna påverkar dig. En annan viktig del i din yogaträning är att lyssna till kroppen. Om en övning gör fysiskt ont, gör den långsammare, kortare tid – eller inte alls! Yoga ska vara roligt – och aldrig göra ont.

Om Yoga

Yoga – ett stort begrepp, som omfattar många olika aspekter av livet och vardagen. Ordet yoga sägs ofta betyda just balans. Det kommer från sanskrit och dess rot är ordet yuj och betyder ordagrant ok eller förening. Yoga syftar bokstavligen till att ta på sig oket, att anstränga och disciplinera sig, att åstadkomma balans mellan kropp och sinne, kropp och själ. Yoga är ett system för fysisk, mental och andlig träning. En vanlig missuppfattning om yoga är att det är ett system för fysiska övningar och ställningar. Faktum är att yoga är en fullständig vetenskap för livet, ett fullständigt system vilket inkluderar fysiska ställningar (asanas) som en av många olika komponenter.

Utövandet av yoga går långt tillbaka. Arkeologiska utgrävningar i Indien och Pakistan daterar yogan minst 4 000-5 000 år tillbaka. Enligt många yogamästare är yoga mycket äldre än så. Länge var yogan förbehållen ett litet fåtal men idag utövas dessa tekniker av många hundra miljoner människor över hela världen. Genom årtusendena har yoga felaktigt kopplats samman med olika teologiska och filosofiska system. Yoga i sig själv är inte någon religion och är inte heller kopplad till någon specifik religion. Yoga är en filosofi som först och främst är en praktisk fysisk, mental och andlig disciplin som förespråkar personligt utövande, ledande till andlig insikt. Yoga är helt enkelt ett verktyg som ger dig möjlighet att utforska din egen mänskliga natur.

Yogan påstår sig inte vara någon slags enda eller rätta väg till frälsning. Yogan utesluter aldrig någon från att delta. Tvärtom – yogaövningar kan utföras – och gör det också, av alla typer av människor över hela världen, oavsett religiös uppfattning och tillhörighet. När du tränar yoga behöver du inte tro på något annat än möjligheten att du genom yogaträningen kan transformera, förändra dig själv i riktning mot din egen fulla potential och komma i kontakt med din egen innersta sanna kärna, den du verkligen är.

Om Yoga

Yogaövningarna som utförs långsamt och kontrollerat är ämnade att utveckla fysisk smidighet, skapa mental avspänning och ge insikt. Andningen spelar en viktig, grundläggande roll i yogan. Enligt den yogiska filosofin förkroppsligar andningen människans livskraft, eller Prana. Hos de flesta människor är andningen en omedveten funktion. Den kan dock ändras medvetet genom användning av olika andningstekniker, vilket i sin tur även påverkar välbefinnandet. Andningen är också en direkt avspegling av olika känslomässiga tillstånd. Är vi uppjagade andas vi snabbt och ytligt. Är vi lugna andas vi djupare, lugnare andetag. Därför kan en medveten förändring av andningen direkt påverka hur vi mår och skapa fysisk, mental och emotionell harmoni inom oss. Andningen är ett unikt sätt att direkt kunna påverka sinnet och kroppens undermedvetna processer.

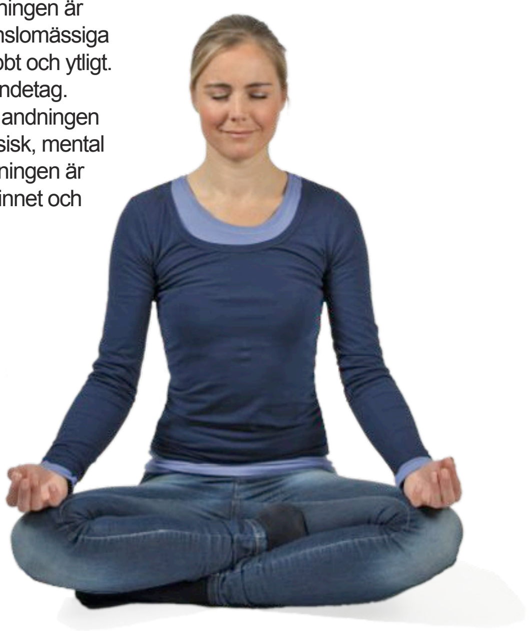

Yoga påverkar till exempel:

- Andning och lungor
- Muskulaturen
- Matsmältningsapparaten
- Körtelsystemet
- Lymfsystemet
- Nervsystemet och hjärnan

Om Meditation

Meditation är en viktig del i MediYoga.
Yogaprogrammet avslutas normalt sett med en meditation och det finns många sätt att meditera.

Kroppens negativa reaktioner på stress minskar. Mätningar av muskelspänningar under meditation har visat att dessa sjunker nästan ner till noll. Det gör att meditation kan lindra högt blodtryck, huvudvärk och kroniska smärttillstånd. Blodcirkulationen förbättras och EEG-mätningar visar att hjärnans aktivitet under meditation liknar den vid sömn vilket kan vara till hjälp för människor med sömnrubbningar. Liksom yoga ger meditation andrum, rum för anden. Meditationen syftar till att stärka viljan och din förmåga att ordna tillvaron på bästa sätt.

Om Meditation

Mer att säga om meditation

- **Meditation ger ökad koncentration och mental snabbhet**
- **Meditation hjälper dig att leva med en större medvetenhet och balans i dig själv**
- **Meditation ger ett inre lugn som hjälper till att utveckla din intuition**
- **Meditation gör dig mindre reaktiv, d v s mindre styrd av enbart dina känslor**

Meditation kräver koncentration, uthållighet och tid, 11 minuter, 1-2 gånger om dagen rekommenderas. De flesta kan lära sig meditera. Till viss del handlar det om att ”städa upp på vinden” – inte alltid så roligt men välbehövligt och skönt när det är gjort.

När du börjar meditera kan du märka hur tankarna och sinnet drar iväg med dig. Du kanske upplever att du är okoncentrerad och det är en helt normal reaktion. Använd mantra, andning eller den teknik du använder i meditationen för att återfå fokus igen. Lägg märke till hur du kan få upp tankar som relaterar till aktuella problem. Den djupt liggande stress som de flesta har inom sig, kan ta form som ytligt liggande tankar.

Det finns mängder av olika sätt att meditera och i grunden handlar det om olika vägar till samma mål. T ex. koncentrations-, andnings-, ljud-, mantra-, visualiserings- och rörelsemeditationer. MediYoga innehåller hundratals olika meditationer för avspänning, balans, depression, energi, hjärta, healing, hjärna, intuition, koncentration, kreativitet, mental kontroll, nervstyrka, självtillit, stress, viljestyrka och vitalitet - för att bara nämna några.

Släpp alla förväntningar när du mediterar
Bevittna
Känn efter
Innerst inne vet du vem du är och vart du är på väg

Det långa djupa andetaget

Det långa djupa, medvetna andetaget, är grunden inom MediYoga

Det långa djupa andetaget består av tre delar:
Magandning. Lägg händerna en bit ner på magen. Andas in genom näsan och styr andetaget ner i buken så att magen putar ut. Hjälp till med magmusklerna så att händerna lyfts mot taket. Fyll på med andetaget i svanken och ut på ryggen. Känn magen. Vid utandning släpper du långsamt magen och andas ut hela vägen. Avsluta med att dra in magen lite grann, så att magen töms på luft.

Revbensandning. Placera händerna mot revbenen, slappna av i skuldrorna. Andas in genom näsan och fyll bröstkorgen ut åt sidorna och sedan ut på ryggen och fram i bröstet. Låt bröstkorgen expandera. Andas ut genom näsan och låt bröstkorgen komma ner. Känn rörelsen under händerna.

Nyckelbensandning. Lägg en hand högt uppe på bröstet så att tumme och pekfinger rör vid nyckelbenen. Andas in djupt, genom näsan, lyft nyckelbenen i en rät linje så att hela bröstkorgen höjs, utan att axlar eller rygg följer med. Andas sedan ut genom näsan och sänk nyckelbenen med bröstkorgen. Känn rörelsen under dina fingrar.

Dessa tre delar sätts samman till ”det långa, djupa, yogiska andetaget”.
Andas in genom näsan, fyll magen som en ballong. Fortsätt så att bröstkorgen går upp och ut åt sidorna. Fortsätt fylla med luft ända upp till nyckelbenen. Gör en naturlig paus och andas sedan ut i omvänd ordning; först sjunker nyckelbenen, därefter bröstkorgen och slutligen sjunker magen ner igen. Låt magen och bröstkorgen vidga sig mer och mer för varje andetag. Ett bra sätt att träna andningen är liggande på rygg. Håll en hand på magen och en på bröstet och känn andningens rörelse. Om ingenting sägs om andningen i en övning så är det långa djupa andetag du ska använda, även mellan övningarna.

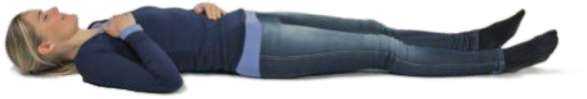

Långa djupa andetag:
- **Skapar lugn och avspänning, ger klarhet och tålamod**
- **Reducerar gifter och slem från lungor och luftvägar**
- **Underlättar blodcirkulationen**
- **Förhindrar ackumulation av kolesterol i blodet**
- **Stimulerar kemisk balans i hjärnan och reglerar pH-värdet**
- **Ökar flödet av spinalvätska till hjärnan**
- **Motverkar depression, osäkerhet och rädsla**
- **Löser upp blockeringar i det praniska flödet**
- **Stärker ditt elektromagnetiska fält, din aura**
- **Påverkar hypofysen, öppnar din intuition m.m**

Suficirklar

6 minuter

Sitt i lätt meditationsställning eller bredbent, långt fram på en stol. Håll händerna på knäna och rotera långsamt bäckenbotten i stora cirklar åt höger, medsols. Svanka fram med höften och låt höften glida ut över höger lår, fortsätt rulla höften bakåt så att du kröker ryggen mjukt, rulla höften vidare ut över vänster lår och svanka fram igen. Föreställ dig att du skruvar ner bäckenbotten i underlaget, djupt ner, varv för varv.

Sträva efter att hålla huvudet centrerat så att det går i motsatt riktning mot rörelsen. När ryggraden rör sig bakåt, strävar sig huvudet framåt, när ryggraden rör sig åt höger går huvudet till vänster osv. På det sättet påverkas hela ryggen. Efter tre minuter byter du riktning och roterar på samma sätt åt andra hållet. Ögonen är slutna, fokus i tredje ögat. Vibrera mentalt mantrat SAT NAM i takt med andetag och rörelse.

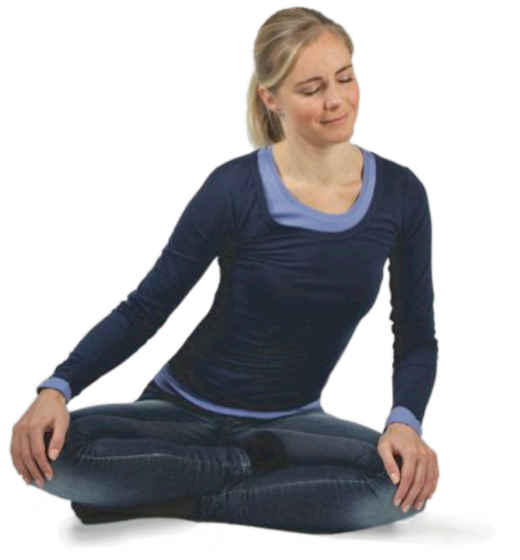

Det här är en roterande övning som mjukar upp rygg och höfter, ökar genomblödningen i hela ryggen, samtidigt som den stimulerar matsmältningen. En mycket grundande övning.

Ryggböjningar

3-9 minuter

1. Sitt i lätt meditationsställning. Vrid överkroppen åt vänster, sätt händerna i golvet på var sida om knäet. Sjunk med rak överkropp ner över vänster ben. Häng och/eller ligg där 1-3 min. och andas långa djupa andetag. Andas sedan in och räta upp överkroppen.
Byt sida, andas ut ner över höger knä.
Häng och/eller ligg där 1-3 min.
Tänk Sat på in- och Nam på utandning.
Andas in, kom upp in i mitten med rak rygg. Ta ett par långa, djupa andetag och centrera dig.

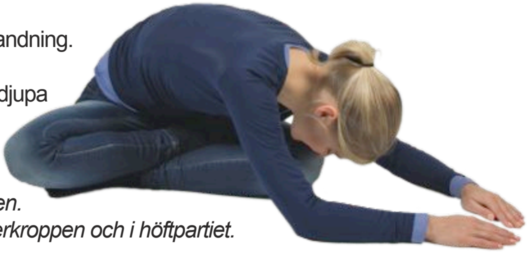

Den här övningen sträcker ut ryggraden och ryggmuskulaturen.
Den ökar flexibiliteten i hela överkroppen och i höftpartiet.

2. Sitt kvar i samma ställning. Sätt händerna i golvet framför knäna. Luta rak överkropp fram ner mellan knäna. Magen först, bröstkorgen sen och huvudet går ner sist. Sträva efter att nå med pannan ända ner i golvet framför dig. När du kommer så djupt fram du kan, stanna där och andas långa djupa andetag. Sat på inandning, Nam på utandning. 1-3 min. Kom sedan, med hjälp av armarna försiktigt, upp ur ställningen. Sitt med rak rygg och ta ett par långa djupa andetag.

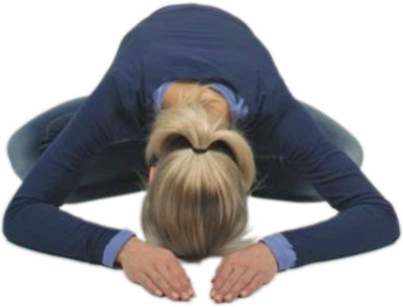

Sträcker ut i ryggen, i höfter, ljumskar och säte.

Yoga för axlar och skuldror

3-9 minuter

Sitt i lätt meditationsställning eller på en stol. Lägg händerna på låren. Andas in, lyft axlarna högt, andas ut, sänk ner dem igen, 1-3 min. Avsluta genom att andas in och dra upp axlarna. Håll. Andas ut och sänk ner dem. Vila.

Mjukar upp i axlar och skulderblad. Balanserar produktionen i sköldkörteln.

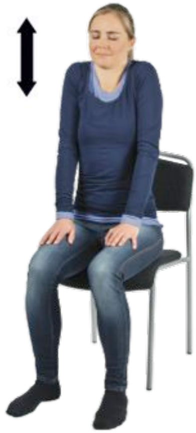

Sitt kvar i samma ställning med händerna på låren. Håll ryggrad och nacke rak och centrerad, det är endast axlar och skuldror som rör sig. Andas in, vänster axel går upp, höger sjunker ner. Andas ut och skifta. Höger axel upp, vänster axel ner, 1-3 min. Vila

Mjukar upp i axlar och skulderblad. Skapar balans mellan höger och vänster hjärnhalva.

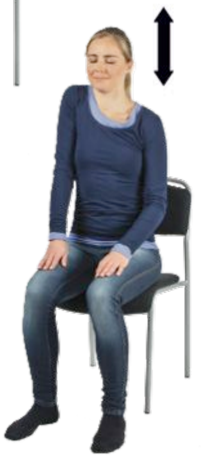

Skulderrullning. Lätt meditationsställning. Lyft upp skuldrorna, dra bak, rulla ner, fram och upp igen, stora cirklar. 1-3 min. Andas in när axlarna går uppåt/bakåt, andas ut när de går neråt/framåt. Vila.

Bra för skuldror, axlar, leder och hjärta. Balanserar sköldkörtel och bisköldkörtel.

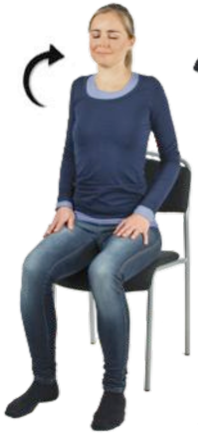

Grundläggande andningsmeditation

11 minuter

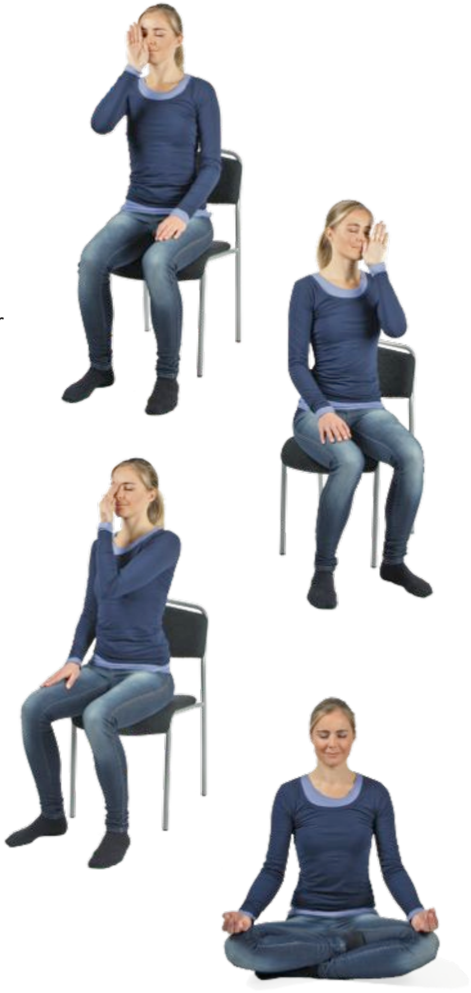

1. Sitt i lätt meditationsställning eller på stol. Sträck höger hands fingrar rakt upp, som en antenn. Tryck tummen mot höger näsborre. Slappna av i axlar och armbåge. Andas långa djupa andetag genom vänster näsborre 1,5 min. Andas in – håll andan kort – ta ner handen igen.

2. Repetera med vänster hand och håll för vänster näsborre. Andas genom höger näsborre 1,5 min. Andas in - håll andan kort och ta sedan ner handen igen.

3. Andas in genom vänster näsborre, andas ut genom höger näsborre. Använd höger tumme och lillfinger för att stänga respektive näsborre 1,5 min.

4. Repetera övning 3 men byt näsborre den här gången. Andas in genom höger näsborre och ut genom vänster näsborre, 1,5 min.

5. Sitt i lätt meditationsställning eller på stol med händerna på knäna och fingrarna i Guyan Mudra, tumme och pekfinger mot varandra, övriga fingrar raka. Koncentrera dig på tredje ögat. Eldandning in och ut genom näsan i 3 min.

6. Slappna av en liten stund med lugna djupa andetag.

Denna andningsmeditation, som kombinerar några olika andningstekniker öppnar dina praniska kanaler och balanserar andningen i kroppen. Den passar bra före ett krävande yogapass. Den är även väldigt bra när du behöver en ”kick” och ett klart, balanserat sinne då den skapar balans i nervsystemet och balans mellan höger och vänster hjärnhalva.

Meditation för starka nerver - lugnt sinne

11-31 minuter

Sitt med rak rygg med benen i kors eller på en stol.

Kvinna: Håll upp vänster hand i höjd med örat, armbågen böjd. Handflatan vänd framåt, fingertopparna pekar uppåt. Tryck mjukt toppen av tummen mot toppen av ringfingret. Höger hand vilar på höger lår med toppen av tummen mjukt tryckt mot lillfingertoppen. Naglarna ska inte röra varandra.

Man: Håll upp höger hand i höjd med örat, armbågen böjd. Handflatan vänd framåt, fingertopparna pekar uppåt. Tryck mjukt toppen av tummen mot toppen av ringfingret. Vänster hand vilar på höger lår med toppen av tummen mjukt tryckt mot lillfingertop-pen. Naglarna ska inte röra varandra.

Andas långa, djupa andetag in och ut genom näsan. Fortsätt i 11-31 minuter.

Avsluta med ett långt, djupt andetag. Lyft båda händer högt över huvudet och skaka händerna kraftfullt under ett par minuter. Ta ner armarna, sitt i stillhet en stund och känn hur det känns.

Vila.

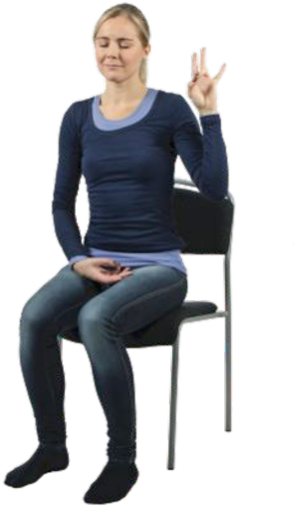

Yogan säger att vi lever i en tid av stora omställningar nu när vi går från Fiskarnas tidsålder in i Vattumannens tidsålder. Många upplever att det är svårt att följa med i de här stora förändringarna. Tillsammans med befolkningsökning och dramatiska klimat-förändringar resulterar det i osäkerhet och instabilitet hos många människor. Den här meditationen är en teknik som ger kraft och balans för att stå stadigt och klara av alla förändringar på ett bra sätt.

Forskning - Utbildning - Kunskap

Några exempel på studier med MediYoga - medicinsk yoga

Förmaksflimmer och MediYoga, 2011
Danderyds Sjukhus, Stockholm

Stroke och MediYoga, 2011-2012
Danderyds Sjukhus, Stockholm

Kroniskt Trötthetssyndrom och MediYoga, 2011-2012
Danderyds Sjukhus, Stockholm

Kroniska ryggproblem/smärtor och MediYoga, 1998 med uppföljning 2011.
Karolinska Sjukhuset, Stockholm

Hjärtsvikt och MediYoga, 2011-2012
Huddinge Sjukhus, Stockholm

Stress och MediYoga, 2011-2012
Nora Vårdcentral/Primärvård
Mediyoga erbjuds till patienter med stressrelaterade sjukdomar och symtom som utbrändhet, oro, ångest, depression och sömnproblem.

Leukemi och MediYoga, 2011-2012
Högskolan i Halmstad
Barn 8-12 år

Högt blodtryck och MediYoga, 2010-2011
Svedala Vårdcentral/Primärvård

MediYoga gör egna studier
Sedan 2010 sammanställer IMY (Institutet för Medicinsk Yoga) resultat från mängder av ”före-och-efter”-enkäter som instruktörer och terapeuter i MediYoga skickar in från sina elever/klienter.

Vill du göra en studie?
Hör av dig till oss på info@mediyoga.se så hjälper vi dig.

MEDIYOGA®

Kraften och verktygen att förändra
ditt öde finns inom dig
