# Supplementary material for: MediYoga compared to physiotherapy treatment as usual for patients with stress-related symptoms in primary care rehabilitation: A randomized controlled trial
Source: PLoS One. 2024 Jun 13;19(6):e0300756. doi: 10.1371/journal.pone.0300756 (PMC11175516; doi:10.1371/journal.pone.0300756)
Supplement: S6 File — (DOCX) [file pone.0300756.s008.docx]

**This is a translation from Swedish to English of the 230411**

**Project Plan / Study Protocol, that is approved by**

*EPN Regional Ethics Review Board in Gothenburg* Dnr: · 658-16 Exp.2016-08-11

Diary number: VGFOUGSB-602221

Project funds - application

Application started by: Madeleine Bel I fiord, 2016-03-04

Occupational title at the time of application: Leg. physiotherapist

Workplace at the time of application: Närhälsan Gibraltar rehab reception

Last modified I corrected by: Madeleine Bellfiord, 2016-03-31

Application submitted to: The R&D Council for Gothenburg and southern Bohuslän

~Decided - granted

Applicant: Madeleine Bellfjord show more info »

Physical therapist, Närhälsan Gibraltar rehab reception

• Applications

• possibly

• Personal data

VGFOUGSB-602221: Evaluation of medical yoga, Mediyoga in group,

compared to usual treatment by a physiotherapist, for people with experienced,

stress-related symptoms. Project funds - application - This application (2016)

~Decided - granted

Basic information

name:

Madeleine Bellfjord

workplace:

Local health Gibraltar rehab clinic

professional title:

Leg. physical therapist

job title:

Physical therapist

Physiotherapy degree

academic degree: Master's degree

Updated 2016-03-04 by Madeleine Bellfjord

Overall project information

The project's scientific area

Medicine

Your form of employment

~Permanent employment in the Västra Götaland region

County council - Västra Götaland region - Community health - Rehabilitation - Area

R lO - Local health Gibraltar rehab reception

How far has the project progressed?

Newly started project (data collection not started)

Part in education?

No part in education

Supervisor Maria Larsson

Operations manager, R&D primary care Gothenburg and Södra Bohuslän

Project content

Estimated project start

2016-09-01

Estimated project end

2017-02-28

**Summary**

Within rehabilitation, there is an increased influx (of people applying for stress-related symptoms). MediYoga in groups is today a sought-after treatment method in rehabilitation. The purpose of this study is to compare the effect of MediYoga in a group, as a treatment method, compared to the effect of usual treatment by a physiotherapist, for people with experienced, stress-related symptoms.

Forty people are randomized to, 1) Intervention group - Mediyoga in a group for eight weeks, 60 minutes per week, guided by a physiotherapist.2) Control group - Usual treatment by a physiotherapist based on a first assessment visiting 60 minutes. Follow-up takes place, after eight and twenty weeks from the baseline measurement, with regard to, self-reported, experienced - stress, anxiety, depression and quality of life. Thorax excursion is measured by an independent physiotherapist for the study.

The study is expected to evaluate whether Mediyoga in a group has a higher stress-reducing effect than usual treatment by a physiotherapist.

**Keyword**

Embedded MeSH terms

**Stress, Psychological (Stress, Psychological)**

Stress wherein emotional factors predominate.

**Anxiety**

Feelings or emotions of dread, apprehension, and impending disaster but not disabling as with ANXIETY DISORDERS.)

**Rehabilitation (Rehabilitation)**

Restoration of human functions to the maximum degree possible in a person or person's suffering from disease or injury.

**Mental Health**

The state in which the person is well adjusted.

**Yoga (Yoga)**

A major orthodox system of Hindu philosophy based on Sankhya (metaphysicaldualism) but differing from it in being theistic and characterized by the teaching of raja-yoga as a practical method of liberating the self. It includes a system of exercises for attaining bodily or mental control and well-being with liberation of the self and union with the universal spirit. (From Webster, 3d ed)

**Complementary Therapies**

Therapeutic practices which are not currently considered an integral part of conventional allopathic medical practice. They may lack biomedical explanations but as they become better researched some (PHYSICAL THERAPY MODALITIES; DIET; ACUPUNCTURE) become widely accepted whereas others (humors, radium therapy) quietly fade away, yet are important historical footnotes. Therapies are termed as Complementary when used in addition to conventional treatments and as Alternative when used instead of conventional treatment.

**Evidence-Based Medicine**

The process of systematically findings and appraising and using contemporaneous research findings and the basis for clinical decisions. Evidence-based medicine asks questions, finds and appraisings the relevant data, and harnesses that information for everyday clinical practice. Evidence-based medicine follows four steps: formulate a clear clinical question from a patient’s problem; search the literature for relevant clinical articles; evaluate (critically appraise) evidence for its validity and usefulness; implement useful findings in clinical practice. The term “ evidence-based medicine” (no hyphen) was coined at Mc Master Medical School in Canada in the 1980´s to label this clinical learning strategy, which people at the school had been developing for a decade (From BMJ 1995;310:1122)

**Primary Health Care**

Care which provides integrated, accessible health care services by clinicians who are accountable for addressing a large majority of personal health care needs, developing a sustained partnership with patients, and practicing in the context of family and community. (JAMA 1995;273(3):192)

**Background**

Mental ill health is a leading cause of long-term sickness absence in Sweden, as in many economically developed countries (1). Health care is still inadequately equipped to meet the need for interventions for common, less serious forms of depression, anxiety and stress-related symptoms (2). Stress as a phenomenon is difficult to define and has always been controversial since it was first described by Selye (1936). Selye meant that stress and stress reactions are one unspecific and general bodily reaction to every kind of stress that we are exposed to (3).

Yoga as a philosophy and form of exercise has existed for about 4-5000 years. The word yoga means union -union of body and soul (4). There are several yoga orientations, common to all of them is that train none, is a combination of physical training, breathing techniques and meditation. "Guidelines for rehabilitation in stress-related health", 2016, shows, limited evidence for yoga (5). Several published studies (6-10) evaluated yoga, mindfulness and meditation and its impact on people with excessive stress and anxiety, shows significant results with reduced perceived stress and anxiety levels.

MediYoga has its origins in Kundalini yoga and is a therapeutically developed form of yoga, with simple, soft exercises, which include physically determined positions and end with meditation. Research into MediYoga has been conducted and is ongoing in Sweden since 1997 (11). In a study published in 2013, significant results were found regarding self-rated stress, anxiety, health-related quality of life and thoracic excursion, after MediYoga training compared with usual treatment in primary care (12). Several rehab clinics within primary care in Gothenburg today offer MediYoga in groups. The treatment method is increasingly in demand by patients. However, there is a lack of studies in the area where MediYoga is compared to usual treatment by a physiotherapist to evaluate how MediYoga affects people with stress and anxiety-related problems.

**Purpose**

The purpose of the study is to compare the effect of MediYoga in a group as a treatment method, compared to the effect of usual treatment by a physiotherapist, for people with stress-related symptoms.

**Question/ Hypothesis/Theoretical frame of reference**

The hypothesis is that MediYoga in a group has a higher stress-reducing effect than usual treatment by a physiotherapist for people with perceived stress- and anxiety-related problems.

**Method:** Selection

Recruitment takes place from people aged 15 and over, who apply to the Gibraltar rehab clinic with self-perceived stress- and anxiety-related complaints. Inclusion criteria: Self-perceived stress and anxiety, including tension in the neck and shoulders, sleep problems, anxiety and headaches pain. Exclusion criteria: not understanding the Swedish language, pregnancy, psychotic elements in the symptom picture, ongoing or having had physiotherapy treatment in the last three months or in MediYoga in a group. Other medical or psychological treatment remains during the study period.

Power has been calculated on the primary outcome measure, the Perceived Stress Scale. With a difference in mean value between intervention group and control group of 7 points and one standard deviation of 7.0, alpha value = 0.05 with a power of 80%, (12) needs 16 people recruited to each group. Since dropouts are anticipated to be 15%–20%, 4 people are recruited further to each group and a total of 40 people are included in the study.

**Method**: Group division

Potential patients for the study are booked in by web or telephone, for an initial assessment visit to a physiotherapist at the Gibraltar rehab reception according to regular routine. The patients who meet the inclusion criteria for the study are informed, both verbally and in writing. Those who wish to participate in the study are informed that she or he will be dialed, for randomization - process.

A person otherwise independent of the study performs randomization by following the patient agreed to participate, draw a sealed non-transparent envelope from a box where the envelopes marked with I (=intervention group) alt K (=control group) have been sorted through a 5 block randomization by a computer-generated randomization program. If the patient is randomized to control group, an appointment is booked with a physiotherapist at the Gibraltar rehab reception. If the patient is randomized to the intervention group, the patient is booked into the group start for MediYoga.

**Method:** Intervention

Intervention group - MediYoga in a group for eight weeks, 60 minutes per week, guided by physiotherapist/project manager, program "Start up package 1", for four weeks and "Start up package 2", during four weeks, prepared by Göran Boll, developer of Mediyoga who form of treatment (11). Control group - Usual treatment by a physiotherapist, based on an initial assessment, one visit in 60 minutes. Usual treatment by a physiotherapist may include, physical exercise, fitness and strength, relaxation, body awareness, counselling, stress management, acupuncture treatment and transcutaneous electrical nerve stimulation, (TENS).

"Guidelines for rehabilitation in stress-related ill health" exists (5). The number of visits to a physiotherapist can vary from one visit for advice, to visits twice a week for example, training over a number of weeks. The time for return visits can vary between 30-45 minutes.

**Method**: Data collection

All estimation forms (entered by the data survey system EsMaker**)** are sent via e-mail to patients in both the intervention group and the control group, one week before the start of treatment.

Thorax excursion, which is measured by a physiotherapist, is collected by a visit to the reception, one week before the start of treatment for both intervention group and control group. This constitutes the baseline data. Follow-up takes place by sending out all estimate forms again after eight and twenty weeks, for primary and secondary outcome measures.

Primary outcome measure, measured with the Perceived Stress Scale, (PSS), (13-14), for perceived stress, secondary outcome measures, measured with the Hospital Anxiety and Depression Scale, (HADS), (15), for perceived symptoms of depression and anxiety and health-related quality of life, measured with Euro Quality of Life VAS, (EQ-VAS), (16). Thorax excursion: Measured in cm, with a measuring tape, the circumference of the chest at the level of the processus xiphoideus, difference between maximal inhalation and exhalation, after eight and twenty weeks. Measurement is performed by a non-treating physiotherapist independent of the study. At the measurement, the patient places both hands on the head and receives the instruction" breathe in maximally and make yourself as big as possible" and "exhale maximally and make yourself as small as possible",(17).

**Method**: Data processing

Descriptive statistics will be used to describe the sample at baseline. Study's hypothesis will be tested with respect to difference between intervention group and control group, as well as in change between baseline and follow-up, within the intervention group respectively within the control group. Intention-to-treat analysis will be used.

For variables with ordinal data: such as, perceived stress, depression and anxiety comes non parametrically statistics to be used such as the Mann-Whitney U test and Wilcoxons sign rank test. For variables with continuous data: such as quality of life and thoracic excursion will parametric statistics to be used, provided the data is normally distributed, such as student's t-test and paired t-test**.**

**Expected result/ Clinical significance**

In rehabilitation and physiotherapy, several treatment methods are used today that are clinically relevant and the patients' experience is often positive. It is of the greatest value that scientifically evaluate the treatment methods that physiotherapists offer, within primary care to patients today. The target group the study is aimed at accounts for an increasing part of sick leave, hence it is especially important to try to find treatments that improve their health. It is also possible that it can be more cost-effective with fewer visits to physiotherapist and the patients hopefully achieve good mental health. MediYoga in a group is a very popular treatment method for people with stress-related symptoms. If MediYoga in a group would prove to be more effective than usual treatment in physiotherapist for people with increased stress, anxiety and worry, physiotherapists can eventually offer and include MediYoga in groups as an evidence-based treatment method within rehabilitation.

References

1. Henderson M, Harvey SB, 0verland S, Mykletun A, Hotopf M. Work and common psychiatric disorders. J R Soc Med 2011;104: 198-207.

2. The insurance fund. Sickness absence in mental health diagnoses. Social security report 2014:4,Försäkringskassan, 2014.

3. Selye, H. A syndrome produced by diverse noxious agents. Journal of Neuropsychiatry Clinical Neuroscience: 1998, 10:2, 230-1. Original edition Nature 138:32, 1936.

4. Papp M.Yoga, effect on heart, vessels, mood and melatonin. Sports Medicine 2008;4:12-16.

5. http://intra.narhalsan.se. Care and Health - Local health medical guidelines-Common guidelines Rehabilitation - Stress-related illness.

6. Michalsen A, Jeitler M, Brunnhuber Set al. "Iyengar yoga for distressed women: a 3-armed randomized controlled trial", Evidence-Based Complementary and Alternative Medicine, vol. 2012, Article ID 408727, 9 pages.

7. Brems C. A Yoga Stress Reduction Intervention for University Faculty, Staff, and GraduatesStudents. International Journal of Yoga Therapy: 2015, Vol. 25, no. 1, pp. 61-77.

8. Barrett CJl. Mindfulness and Rehabilitation: Teaching Yoga and Meditation to Young Men in an Alternative to Incarceration Program. Int J Offender Ther Comp Criminol. 2016 Feb 22.

9. Dos Santos TM1, Kozasa EH2, Carmagnani IS3, Tanaka LH4, Lacerda SS5, Nogueira Martins. Positive Effects of a Stress Reduction Program Based on Mindfulness Meditation in Brazilian Nursing Professionals: Qualitative and Quantitative Evaluation. LAl. 2016 MarApr;12(2):90-9. Epub 2015 Dec 17.

10. Yoga for Adult Women with Chronic PTSD: A Long-Term Follow-Up Study. Rhodes Al ,2, SpinazzolaJl, van der Kolk Bl. J Altern Complement Med. 2016 Mar;22(3):189-96.

11. www.mediyoga.se

12. Köhn M, Persson Lundholm U, Bryngelsson I-L, Anderzen-Carlsson A, Westerdahl E. Medical Yoga for Patients with Stress-Related Symptoms and Diagnosis in Primary Health Care: A Randomized Controlled Trial. Evidence-Based Complementary and Alternative Medicine 2013.

13. Cohen S, Kamarck T, Mermelstein RA. Global Measure of Perceived Stress, J Health Soc Behavior 1983;24(4):385-396.

14. Eklund M, Bäckström M, Tuvesson H. Psychometric properties and factor structure of the Swedish version of the Perceived Stress Scale. Nord J Psychiatry. 201 4;68(7) :494-9.

15. Zigmond AS, Snaith RP. The Hospital Anxiety And Depression Scale, Acta Psychiatrica Scandinavica 1983;67:361-370.

16. EuroQol Group, "EuroQol: a new facility for the measurement of health-related quality of life," Health Policy, 1990;16(3): 199-208.

17. Olsen F. M, Lindstrand J, Broberg L, and Westerdahl E. "Measuring chest expansion, a study comparing two different instructions," Advances in Physiotherapy. 2011; 13: 128-132.

**Ethical examination**

The application to the ethics review board has been made or is planned

Any other attachments

Appendices

Appendix 1. Start up package 1

Appendix 2 Start up package 2

Requested funds/Total budget

Staff -

Project manager

Staff -

Brief description of cost

Physiotherapist 27% during 6 months

SEK 32100 times 0.27 = 8667 times 1.45 times 6 months=

SEK 75,402

Physiotherapist, who performs measurements, of 40 people at three

Amount

75,402

29,362

Assisting staff

occasions, baseline, after 8 weeks or 20 weeks. Can measure

four-person, thoracic excursion, in one hour.

120 measurements for 40 people, a total of 30 hours for measurements.

30 hours, 75% of monthly salary SEK 27,000 times 1.45 = SEK 29,362

Randomization by telephone. Can perform 4 randomization calls

per hour, a total of 10 hours for 40 people.

A few extra calls may be needed if the dropout happens immediately

telephone is added 2 hours extra to a total of 12 hours.

Monthly salary 25000 times 0.12 times 1.45 = SEK 4350.

Total 109114

~Decision

Decision date: 2016-04-28

Brief description of each Claimant 1

' Decision

Comment on decision

cost means SEK

**Personnel - Project manager**

Physiotherapist 27% during 6 months SEK 321 times 0.27 = 8667 times

1.45 times 6 months = SEK 75,402

**Personnel - Employees**

Physiotherapist, who performs measurements, of 40 people on three occasions, baseline, after 8 weeks or 20 weeks. Can measure four people's in one hour.

120 measurements for 40 people, becomes a total of 30 hours for measurements.

30 hours, 75% of monthly salary SEK 27,000 times 1.45 = SEK 29,362

**Staff - Assisting staff**

Randomization by telephone. Can perform 4 randomization calls per

hour, a total of 10 hours for 40 people.

Then some extra calls may be needed If you miss out, the phone will be added immediately

2 extra hours to a total of 12 hours.

Monthly salary 25000 times 0.12

times 1.45=SEK 4350. 4,350 4,236

sum 109114 109,000

Decision on ethical review

Ethical examination is required for disbursement of funds

Comment on decision

Unclear what the control group gets. Note what they get and record it.

NOTE: When requisitioning granted funds; don't forget to submit a copy of ethical approval

if this has not already been done at the time of application.

Consumption period

Last day of use of allocated funds: 2018-04-28

.
