# Supplementary material for: MediYoga compared to physiotherapy treatment as usual for patients with stress-related symptoms in primary care rehabilitation: A randomized controlled trial
Source: PLoS One. 2024 Jun 13;19(6):e0300756. doi: 10.1371/journal.pone.0300756 (PMC11175516; doi:10.1371/journal.pone.0300756)
Supplement: Supplementary file 6 [file pone.0300756.s006.docx]

Regionala etikprövningsnämnden i Göteborg

Projektansvarig: Maria Larsson
Västra Götalandsregionen
Närhälsan FoU primärvård
Kungsgatan 12, vån 6
411 18 Göteborg

Dnr: 658-16

Exp. 2016-08-11

Forskningshuvudman: Västra Götalandsregionen

Närvarande beslutande:
Bo Rolfson, *ordförande*
Bibbi Ringsby-Jansson, *bitr. vetenskaplig sekreterare (deltog ej i ärende 486-16 p.g.a. jäv)*
Ledamöter med vetenskaplig kompetens:
Jesper Lundgren *(vetenskaplig sekreterare ärende 486-16)*
Johan Berlin
Elisabeth Björk Brämberg
Karin Klinga Levan
Peter Korp *(deltog ej i ärende 633-16 p.g.a. jäv)*
Anna Nordenstam
Ann Svensson
Helle Wijk
Ledamöter som företräder allmänna intressen:
Bengt Fernström
Beatrice Toll
Ingmarie Torstensson
Robert Engström

Projekttitel: Utvärdering av medicinsk yoga, Mediyoga i grupp, jämfört med sedvanlig behandling hos fysioterapeut, för personer med upplevd stressrelaterad ohälsa
Projekt ID: VGFOUGSB-602221

Beslutsprotokoll från sammanträde med Regionala etikprövningsnämnden i Göteborg, Avdelningen för övrig forskning, den 8 augusti 2016

Föredragande: Elisabeth Björk Brämberg

Godkännes med villkor

Etikprövningsnämnden godkänner studien med följande villkor:

- *att* gruppen forskningspersoner avgränsas till de som är 18 år eller äldre, då frågeformulären innefattar känsliga frågor och det kan vara svårt för yngre att ta ställning till deltagande i en studie som syftar till att prova olika behandlingsalternativ för stress,

- *att* samtyckesblanketten kompletteras på så sätt att forskningspersonerna ges möjlighet att samtycka till att uppgifter, i förekommande fall, kan hämtas från deras journal, och
- *att* det finns möjlighet till och information om uppföljning inom vården, i de fall forskningspersoner i studien visar tecken på depression eller liknande.

Beslutet kan överklagas till Centrala etikprövningsnämnden. Skrivelsen ska vara ställd till Centrala etikprövningsnämnden men skickas eller lämnas till Regionala etikprövningsnämnden i Göteborg. Överklagandet ska vara underskrivet av behörig företrädare för forskningshuvudmannen. Denne kan lämna fullmakt till forskaren att skriva under. Bifoga i så fall fullmakten. I skrivelsen ska anges vilket beslut som överklagas och den ändring som Ni begär. Överklagandet ska ha kommit in till Regionala etikprövningsnämnden senast tre veckor från det att klaganden fick del av beslutet.

Att denna avskrift i transumt överensstämmer med originalet intygar:

Barbro Morsing, administrativ sekreterare
