# Supplementary material for: MediYoga compared to physiotherapy treatment as usual for patients with stress-related symptoms in primary care rehabilitation: A randomized controlled trial
Source: PLoS One. 2024 Jun 13;19(6):e0300756. doi: 10.1371/journal.pone.0300756 (PMC11175516; doi:10.1371/journal.pone.0300756)
Supplement: Supplementary file 7 [file pone.0300756.s007.docx]

INKOM
2016 -07- 08
Etikprövningsnämnden i Göteborg

Utvärdering av medicinsk yoga, Mediyoga i grupp, jämfört med sedvanlig behandling hos fysioterapeut, för personer med upplevd, stressrelaterad ohälsa.

Diarienummer: VGFOUGSB-602221
Projektmedel - ansökan
Ansökan påbörjad av: Madeleine Bellfjord, 2016-03-04
Yrkestitel vid ansökningstillfället: Leg.sjukgymnast
Arbetsplats vid ansökningstillfället: Närhälsan Gibraltar rehabmottagning
Senast ändrad / åtgärdad av: Madeleine Bellfjord, 2016-03-31
Ansökan inkommen till: FoU-rådet för Göteborg och södra Bohuslän
Beslutad - beviljad
Sökanden: **Madeleine Bellfjord** visa mer info »
Sjukgymnast, Närhälsan Gibraltar rehabmottagning

- Ansökningar
- CV
- Personuppgifter

Madeleine Bellfjord har medverkat i 3 ansökningar till FoU-rådet för Göteborg och södra Bohuslän under de senaste 5 åren

VGFOUGSB-602221 : Utvärdering av medicinsk yoga, Mediyoga i grupp, jämfört med sedvanlig behandling hos fysioterapeut, för personer med upplevd, stressrelaterad ohälsa., *Projektmedel - ansökan* - **Denna ansökan** (2016)
Beslutad - beviljad

Basuppgifter

namn:
Madeleine Bellfjord
arbetsplats:
Närhälsan Gibraltar rehabmottagning
yrkestitel:
Leg.sjukgymnast
tjänstetitel:
Sjukgymnast

Utbildning

högskole- eller universitetsexamen:
Sjukgymnastexamen
akademisk grad:
Magisterexamen

uppdaterad 2016-03-04 av Madeleine Bellfjord

Övergripande projektinformation

Projektets vetenskapsområde

☒Medicin
☐Odontologi
☒Vårdvetenskap

Din anställningsform

☒Tillsvidareanställning i Västra Götalandsregionen
☐Tillsvidareanställning i privat verksamhet med avtal

Arbetsgivare

visa mer info »
Inlagda arbetsplatser

Landsting - Västra Götalandsregionen - Närhälsan - Rehabilitering - Område R10 - Närhälsan Gibraltar rehabmottagning

Aktuell arbetstid

30

Hur långt har projektet framskridit?

Nystartat projekt (datainsamling ej påbörjad)

Del i utbildning

Ej del i utbildning

Medarbetare / Handledare

Handledare

till personkort » **Maria Larsson** visa mer info »
Verksamhetschef, FoU primärvård Göteborg och Södra Bohuslän

- Din webbläsare kan inte hämta CV automatiskt. Klicka här för att gå direkt till CV i personkortet -

Projektets innehåll

Beräknad projektstart

2016-09-01

Beräknat projektslut

2017-02-28

Sammanfattning

Inom rehabilitering sker ett ökat inflöde av personer som söker för stressrelaterad ohälsa. Mediyoga i grupp är idag, en efterfrågad behandlingsmetod inom rehabilitering.
Syftet med denna studie är att jämföra effekten av Mediyoga i grupp, som behandlingsmetod, jämfört med effekten av sedvanlig behandling hos fysioterapeut, för personer med upplevd, stressrelaterad ohälsa.
Fyrtio personer randomiseras till, 1) Interventionsgrupp - Mediyoga i grupp under åtta veckor, 60 minuter per vecka, med guidning av fysioterapeut.
2) Kontrollgrupp - Sedvanlig behandling hos fysioterapeut utifrån ett första bedömningsbesök på 60 minuter.
Uppföljning sker, efter åtta och tjugo veckor från baseline mätning, med avseende på, självrapporterad, upplevd - stress, ångest, depression och livskvalitet. Thorax excursion mäts av för studien oberoende fysioterapeut.
Studien förväntas utvärdera om Mediyoga i grupp har en högre stressreducerande effekt än sedvanlig behandling hos fysioterapeut.

Nyckelord

visa mer info »

Inlagda MeSH-termer

Stress, psykologisk (Stress, Psychological)
Stress wherein emotional factors predominate.

Ångest (Anxiety)
Ständig känsla av oro, ängslan och överhängande faror.
(Feeling or emotion of dread, apprehension, and impending disaster but not disabling as with ANXIETY DISORDERS.)

Rehabilitering (Rehabilitation)
Restoration of human functions to the maximum degree possible in a person or persons suffering from disease or injury.

Psykisk hälsa (Mental Health)
The state wherein the person is well adjusted.

Yoga (Yoga)
A major orthodox system of Hindu philosophy based on Sankhya (metaphysical dualism) but differing from it in being theistic and characterized by the teaching of raja-yoga as a practical method of liberating the self. It includes a system of exercises for attaining bodily or mental control and well-being with liberation of the self and union with the universal spirit. (From Webster, 3d ed)

Alternativ- och komplementärmedicin (Complementary Therapies)
Samlingsbegrepp för ickekonventionella, medicinska behandlingsmetoder, ofta med andra förklaringsmodeller än dem som ges inom den naturvetenskapligt baserade medicinen. Vissa former har efterhand nått erkännande (sjukgymnastik; dietterapi; akupunkturbehandling), medan andra försvunnit och förpassats till den medicinska historien (humoral terapi; radiumbehandling).
(Therapeutic practices which are not currently considered an integral part of conventional allopathic medical practice. They may lack biomedical explanations but

as they become better researched some (PHYSICAL THERAPY MODALITIES; DIET; ACUPUNCTURE) become widely accepted whereas others (humors, radium therapy) quietly fade away, yet are important historical footnotes. Therapies are termed as Complementary when used in addition to conventional treatments and as Alternative when used instead of conventional treatment.)

Bevisbaserad sjukvård (Evidence-Based Medicine)
Systematiskt sökande, utvärdering och utnyttjande av moderna forskningsresultat som underlag för beslut i den kliniska verksamheten. Den bevisbaserade sjukvården följer fyra steg: en klinisk, patientrelaterd frågeställning, sökande efter relevant, kliniskt inriktad litteratur, kritisk värdering av giltighet och nytta av dokumenterade resultat, och omsättande av dessa i klinisk praxis. Begreppet "bevisbaserad sjukvård" (evidence based medicine) myntades vid McMaster Medical School i Kanada på 1980-talet. (The process of systematically finding, appraising, and using contemporaneous research findings as the basis for clinical decisions. Evidence-based medicine asks questions, finds and appraises the relevant data, and harnesses that information for everyday clinical practice. Evidence-based medicine follows four steps: formulate a clear clinical question from a patient's problem; search the literature for relevant clinical articles; evaluate (critically appraise) the evidence for its validity and usefulness; implement useful findings in clinical practice. The term "evidence based medicine" (no hyphen) was coined at McMaster Medical School in Canada in the 1980's to label this clinical learning strategy, which people at the school had been developing for over a decade. (From BMJ 1995;310:1122))

Primärvård (Primary Health Care)
Care which provides integrated, accessible health care services by clinicians who are accountable for addressing a large majority of personal health care needs, developing a sustained partnership with patients, and practicing in the context of family and community. (JAMA 1995;273(3):192)

Bakgrund

Psykisk ohälsa är den främsta orsaken till en längre tids sjukfrånvaro i Sverige, liksom i många ekonomiskt utvecklade länder (1). Hälso-sjukvården är fortfarande otillräckligt rustad för att möta behoven av insatser för vanliga, mindre allvarliga former av depressioner, ångest och stresstillstånd (2). Stress som företeelse är svårdefinierat och har varit kontroversiellt allt sedan det först beskrevs av Selye (1936). Selye menade att stress och stressreaktioner är en ospecifik och allmängiltig kroppslig reaktion på varje slags påfrestning som vi utsätts för (3).

Yoga som filosofi och träningsform har funnits i ca 4-5000 år. Ordet yoga betyder förening – förening av kropp och själ (4). Det finns ett flertal, yogainriktningar, gemensamt för alla är att träningen, är en kombination av fysisk träning, andningsteknik och meditation.
"Riktlinjer för rehabilitering vid stressrelaterad ohälsa", 2016, visar, begränsad evidens för yoga (5).
Ett flertal, publicerade studier (6-10), där man utvärderat yoga, mindfulness och meditation och dess påverkan hos personer med förhöjd stress och ångest, visar på signifikanta resultat med sänkt upplevd stress och ångestnivå.
Mediyoga har sitt ursprung i Kundaliniyoga och är en terapeutisk utvecklad form av yoga, med enkla, mjuka övningar, där fysiska bestämda positioner ingår och avslutas med meditation. Forskning inom Mediyoga har bedrivits och pågår i Sverige sedan 1997 (11).

I en studie publicerad 2013, fann man signifikanta resultat, avseende självskattad -stress, ångest, hälsorelaterad livskvalitet och thorax exkursion, efter träning av Mediyoga jämfört med sedvanlig behandling inom primärvården (12).

Flera rehabmottagningar inom primärvården i Göteborg erbjuder idag Mediyoga i grupp. Behandlingsmetoden blir allt mer efterfrågad av patienter. Det saknas dock studier inom området där Mediyoga jämförs med sedvanlig behandling hos fysioterapeut för att utvärdera hur Mediyoga påverkar personer med stress- och ångestrelaterade besvär.

Syfte

Syftet med studien är att jämföra effekten av Mediyoga i grupp, som behandlingsmetod, jämfört med effekten av sedvanlig behandling hos fysioterapeut, för personer med stressrelaterad ohälsa.

Frågeställning / Hypotes /Teoretisk referensram

Hypotesen är att Mediyoga i grupp, har en högre stressreducerande effekt än sedvanlig behandling hos fysioterapeut för personer med upplevda stress - och ångestrelaterade besvär.

Metod: Urval

Rekrytering sker av personer från 15 år och uppåt, som söker till Gibraltar rehabmottagning med självupplevda stress- och ångestrelaterade besvär.
Inklusionskriterier: Självupplevd stress och ångest, även såsom spänningar i nacke och axlar, sömnbesvär, oro och huvudvärk.
Exklusionskriterier: ej förstår svenska språket, graviditet, psykotiska inslag i symptombilden, pågående, eller ha haft fysioterapeutisk behandling, de senaste tre månaderna. Ej tidigare deltagit i Mediyoga i grupp. Annan medicinsk eller psykologisk behandling kvarstår under studieperioden.

Power har beräknats på primärt utfallsmått, Perceived Stress Scale. Med en skillnad i medelvärde mellan interventionsgrupp och kontrollgrupp på 7 poäng och en standardavvikelse på 7.0, alfa värde = 0.05 med en power på 80%, (12) behöver 16 personer rekryteras till varje grupp. Då bortfall med 15-20% kalkyleras rekryteras 4 personer ytterligare till varje grupp och totalt inkluderas 40 personer i studien.

Metod: Gruppindelning

Potentiella patienter till studien bokas in via web eller telefon, till ett första bedömningsbesök till någon sjukgymnast på Gibraltar rehabmottagning enligt ordinarie rutin.
De patienter som uppfyller inklusions - kriterier för studien informeras, både muntligt och skriftligt. De som önskar delta i studien, informeras om att hon eller han kommer bli uppringd, för randomiserings - process.
En för studien i övrigt oberoende person utför randomisering genom att efter att patienten tackat ja till att delta, dra ett igenklistrat ej genomsynbart kuvert från en box där kuverten märkt med I (=interventiosgrupp) alt K (=kontrollgrupp) har sorterats in genom en

blockrandomisering via ett datorgenerat slumpprogram. Om patienten randomiseras till kontrollgrupp bokas tid in till någon fysioterapeut på Gibraltar rehabmottagning. Om patienten randomiseras till interventionsgrupp, bokas patienten in till gruppstart för Mediyoga.

Metod: Intervention

Interventionsgrupp - Mediyoga i grupp under åtta veckor, 60 minuter per vecka, guidning av fysioterapeut/projektledare, program "Startpaket 1" (bil 1), under fyra veckor och "Startpaket 2", (bil 2), under fyra veckor, utarbetade av Göran Boll, utvecklare av Mediyoga som behandlingsform (11).
Kontrollgrupp - Sedvanlig behandling hos fysioterapeut, utifrån en första bedömning, ett besök på 60 minuter.
Sedvanlig behandling hos fysioterapeut kan innehålla, fysisk träning, kondition och styrka, avspänning, kroppskännedom, rådgivning, stresshantering, akupunkturbehandling och transkutan elektrisk nervstimulering, (TENS). "Riktlinjer för rehabilitering vid stressrelaterad ohälsa" finns (5).
Antal besök hos fysioterapeut kan variera från ett besök, för rådgivning, till besök två gånger i veckan för exempelvis träning under ett antal veckor. Tiden vid återbesök kan variera mellan 30-45 minuter.

Metod: Datainsamling

Samtliga skattningsformulär (som lagts in via dataenkätsystemt EsMaker) skickas via e-post till patienter både i interventionsgrupp och kontrollgrupp, en vecka före behandlingsstart. Thorax excursion som mäts av fysioterapeut, samlas in via besök på mottagningen, en vecka före behandlingsstart för både interventionsgrupp och kontrollgrupp. Detta utgör baseline data.
Uppföljning sker genom att samtliga skattningsformulär åter skickas ut efter åtta och tjugo veckor, för primärt och sekundära utfallsmått.
Primärt utfallsmått, mäts med Perceived Stress Scale, (PSS), (13-14), för upplevd stress, Sekundära utfallsmått, mäts med Hospital Anxiety and Depression Scale, (HADS), (15), för upplevda symptom på depression och ångest och Hälsorelaterad livskvalitet, mäts med Euro Quality of Life VAS, (EQ-VAS), (16).
Thorax excursion: Mäts i cm, med måttband, bröstkorgens omfång i nivå med processus xifoideus, differens mellan maximal inandning och utandning, efter åtta och tjugo veckor. Mätning utförs av icke behandlande och för studien oberoende fysioterapeut. Vid mätningen får patienten placera båda händerna på huvudet och får instruktionen " andas in maximalt och gör dig så stor som möjligt" och " andas ut maximalt och gör dig så liten som möjligt",(17).

Metod: Databearbetning

Deskriptiv statistik kommer att användas för att beskriva stickprovet vid baseline. Studiens hypotes kommer att testas med avseende på skillnad mellan interventions grupp och kontrollgrupp, samt i förändring mellan baseline och uppföljning, inom interventionsgrupp respektive inom kontrollgrupp. Intention-to-treat analys kommer användas.
För variabler med ordinaldata: som, upplevd stress, depression och ångest kommer icke-parametrisk statistik att användas som tex Mann-Whitney U test och Wilcoxons teckenrangtest.

För variabler med kontinuerliga data: som livskvalitet och thorax excursion kommer parametrisk statistik att användas, förutsatt att data är normalfördelad, som tex, student´s t-test och parat t-test .

Förväntat resultat / Klinisk betydelse

Inom rehabilitering och fysioterapi används idag flera behandlingsmetoder som är kliniskt relevanta och patienternas upplevelse är ofta positiv. Det är av största värde att vetenskapligt utvärdera de behandlingsmetoder som fysioterapeuter erbjuder, inom primärvården till patienter idag.
Den målgrupp studien riktar sig till, står för en ökande del av sjukskrivningarna, därav är det särskilt viktigt att försöka finna behandlingar som förbättrar deras hälsa.
Det är också möjligt att det kan vara mer kostnadseffektivt med färre antal besök hos fysioterapeut och patienterna uppnår förhoppningsvis en god psykisk hälsa.

Mediyoga i grupp är mycket efterfrågad behandlingsmetod för personer med stressrelaterad ohälsa.
Om Mediyoga i grupp skulle visa sig vara mer effektiv än sedvanlig behandling hos fysioterapeut för personer med ökad stress, ångest och oro kan fysioterapeuter på sikt erbjuda och inkludera Mediyoga i grupp som en evidensbaserad behandlingsmetod inom rehabilitering.

Referenser

1. Henderson M, Harvey SB, Øverland S, Mykletun A, Hotopf M. Work and common psychiatric
disorders. J R Soc Med 2011;104:198–207. DOI 10.1258/jrsm.2011.100231
2. Försäkringskassan. Sjukfrånvaro i psykiska diagnoser. Socialförsäkringsrapport 2014:4, Försäkringskassan, 2014.
3. Selye, H. A syndrome produced by diverce noucious agents. Journal of Neuropsychiatry Clinical Neuroscience: 1998,10:2, 230-1. Orginalutgåva Nature 138:32,1936.
4. Papp M.Yoga, effekt på hjärta, kärl, humör och melatonin. Idrottsmedicin 2008;4:12-16.
5. http://intra.narhalsan.se. Vård och Hälsa - Närhälsans medicinska riktlinjer-Gemensamma riktlinjer Rehabilitering- Stressrelaterad ohälsa.
6. Michalsen A, Jeitler M, Brunnhuber S et al. "Iyengar yoga for distressed women: a 3-armed randomized controlled trial", Evidence-Based Complementary and Alternative Medicine, vol. 2012, Article ID 408727, 9 pages.
7. Brems C. A Yoga Stress Reduction Intervention for University Faculty, Staff, and Graduate Students. International Journal of Yoga Therapy: 2015, Vol. 25, No. 1, pp. 61-77.
8. Barrett CJ1. Mindfulness and Rehabilitation: Teaching Yoga and Meditation to Young Men in an Alternative to Incarceration Program. Int J Offender Ther Comp Criminol. 2016 Feb 22.
9. Dos Santos TM1, Kozasa EH2, Carmagnani IS3, Tanaka LH4, Lacerda SS5, Nogueira-Martins. Positive Effects of a Stress Reduction Program Based on Mindfulness Meditation in Brazilian Nursing Professionals: Qualitative and Quantitative Evaluation. LA1. 2016 Mar-Apr;12(2):90-9. Epub 2015 Dec 17.
10. Yoga for Adult Women with Chronic PTSD: A Long-Term Follow-Up Study.

Rhodes A1,2, Spinazzola J1, van der Kolk B1. J Altern Complement Med. 2016 Mar;22(3):189-96.
11. www.mediyoga.se
12. Köhn M, Persson Lundholm U, Bryngelsson I-L, Anderzén-Carlsson A, Westerdahl E. Medical Yoga for Patient with Stress-Related Symptoms and Diagnosis in Primary Health Care: A Randomized Controlled Trial. Evidence-Based Complementary and Alternative Medicine 2013.
13. Cohen S, Kamarck T, Mermelstein RA. Global Measure of Perceived Stress, J Health Soc Behavior 1983;24(4):385-396.
14. Eklund M, Bäckström M, Tuvesson H. Psychometric properties and factor structure of the Swedish version of the Perceived Stress Scale. Nord J Psychiatry. 2014;68(7):494-9.
15. Zigmond AS, Snaith R.P. The Hospital Anxiety And Depression Scale, Acta Psychiatrica Scandinavica 1983;67:361-370.
16. EuroQol Group, "EuroQol: a new facility for the measurement of health-related quality of life," Health Policy, 1990;16(3):199-208.
17. Olsén F. M, Lindstrand J, Broberg L, och Westerdahl E. " Measuring chest expansion, a study comparing two different instructions," Advances in Physiotherapy. 2011;13:128-132.

Nödvändiga Bilagor

Etisk prövning

Ansökan till etikprövningsnämnden är gjord eller planeras
Diarienummer på beslut från etikprövningsnämnden
Godkännande från etikprövningsnämnd krävs efter beslut för utbetalning av medel

---

Eventuella övriga bilagor

Bilagor

Startpaket_1.pdf
Bilaga 1. Startpaket 1
Filstorlek: 4032 kB
Startpaket 2 nya.pdf
Startpaket 2
Filstorlek: 4069 kB

Äskade medel/Total budget

Beskrivning	Kort beskrivning av kostnad	Summa
**Personal - Projektledare**	Fysioterapeut 27% under 6 månader<br>32100kr gånger 0.27 = 8667 gånger 1.45 gånger 6 mån = 75402kr	75 402
**Personal -**	Fysioterapeut, som utför mätningar, på 40 personer vid tre	29 362

**Medarbetare**	tillfällen, baseline, efter 8 veckor resp 20 veckor. Kan mäta fyra personers, thorax omfång på en timme. 120 mätningar för 40 personer, blir totalt 30 timmar för mätningar. 30 timmar, 75% av månadslön 27000kr gånger 1.45= 29362kr	
**Personal - Assisterande personal**	Randomisering per telefon. Kan utföra 4 randomiserings samtal per timme, totalt 10 timmar för 40 personer. Då några extra samtal kan behövas om bortfall blir direkt i telefon läggs 2 timmar extra till totalt 12 timmar. Månadslön 25000 gånger 0.12 gånger 1.45=4350kr.	4 350
**Summa**		**109 114**

Beslut

Beslutsdatum: 2016-04-28

Kort beskrivning av respektive kostnad	Äskade medel	Beslut SEK	Kommentar till beslut
**Personal - Projektledare**			
Fysioterapeut 27% under 6 månader<br><br>32100kr gånger 0.27 = 8667 gånger 1.45 gånger 6 mån = 75402kr	75 402	75 402	
**Personal - Medarbetare**			
Fysioterapeut, som utför mätningar, på 40 personer vid tre tillfällen, baseline, efter 8 veckor resp 20 veckor. Kan mäta fyra personers, thorax omfång på en timme.<br>120 mätningar för 40 personer, blir totalt 30 timmar för mätningar.<br>30 timmar, 75% av månadslön 27000kr gånger 1.45= 29362kr	29 362	29 362	
**Personal - Assisterande personal**			
Randomisering per telefon. Kan utföra 4 randomiserings samtal per timme, totalt 10 timmar för 40 personer.<br>Då några extra samtal kan behövas om bortfall blir direkt i telefon läggs 2 timmar extra till totalt 12 timmar.<br>Månadslön 25000 gånger 0.12 gånger 1.45=4350kr.	4 350	4 236	
**summa**	**109 114**	**109 000**	

Beslut om etisk prövning
