# Supplementary material for: MediYoga compared to physiotherapy treatment as usual for patients with stress-related symptoms in primary care rehabilitation: A randomized controlled trial
Source: PLoS One. 2024 Jun 13;19(6):e0300756. doi: 10.1371/journal.pone.0300756 (PMC11175516; doi:10.1371/journal.pone.0300756)
Supplement: Attachment — Submitted filename: 23 nov PLOS Response to reviewers.docx [file pone.0300756.s009.docx]

**Response to reviewers**:

**. Review Comments to the Author**

Please use the space provided to explain your answers to the questions above. You may also include additional comments for the author, including concerns about dual publication, research ethics, or publication ethics. (Please upload your review as an attachment if it exceeds 20,000 characters)

**Reviewer #1:** A two-arm randomized clinical trial was conducted which aimed to compare the effect of MediYoga to conventional treatment for people with perceived stress-related symptoms. For the primary outcome, Perceived Stress Scale, the MediYoga group showed an advantage over physiotherapy, but the difference did not reach statistical significance. As for secondary outcomes, the group effect over time was statistically significant in HADS anxiety and EQ-VAS.

Minor revisions:
1- In the abstract, briefly identify the statistical testing methods used to estimate the p-values. Thank you for your comment, see page 1, line number 28, Mixed effect models was used for analyse.
2- Table 1: For age, provide the mean, standard deviation, and range.

Thank you for noticing this, we have made a new table 1 that includes mean, standard deviation and range, page 5, line number 123. For sex, in addition to the frequencies, provide the corresponding percentages. Thank you for your comment. We choose to present as shown in table 1. There were only two men who participated in the study and with such a limited number of men we chose not to present the number of men in a percentage.
3- Table 3: In addition to the frequencies, provide the corresponding percentages. Thank you for your comment, see table 3 we have added the percentages, page 9, line number 235.
4- Page 9: Statistical Power Calculation: State the statistical testing method which attains 80% power. Perhaps it is a t-test. Yes, the statistical power calculation was done with a t-test.
5- Page 9: The t-test is denoted with lower case t. Thank you for your comment, see page 8 line number 204.
6- Page 9: Perhaps “t-test 2 signed” is a typo for “two-sided t-test.” Thank you for your comment, see page 8 line number 204.
7- Page 9: Indicate the underlying covariance structure used in the mixed-effects linear models. Thank you for your comment, see page 8 line 206. The covariance structure used in the mixed effects model is the variance component for the random effects. For the residuals it is the diagonal.

8- Table 5: Clarify if CI represents 95% CI. Thank you for your comment, completions are made see Table 5, see page 12, line number 258.
9- Table 5: Merge the cells for 8-week follow-up and 20 week follow-up, separately, in the first row. Use the same cell merging format that has been applied to the Baseline section. Thank you for your comment, completions are made see Table 5, page 12 line number 258.
10**-** Clearly describe the results of significant interaction effects

Thank you for your comment. The text in the result section is now adjusted and we hope this clarifies the interaction effect., see page 10 line 247.

“For the secondary outcomes the interaction terms, was statistically significant for HADS anxiety (*P* = 0.01) and EQ-VAS (*P* = 0.03). The eight-week result showed that the MY group decreased in anxiety to a larger extent than the PT group, even the level of anxiety was similar at 20 weeks. For EQ5D the MY group increased to a larger extent than the PT group both at eight and twenty weeks, Table 5.”

HAD Anxiety

|  | Medi Yoga | Physio Therapy |
| --- | --- | --- |
| 0 | 12.7 | 10.8 |
| 8 | 7.3 | 9.3 |
| 20 | 8.7 | 8.8 |

EQ5D VAS

|  | Medi Yoga | Physio Therapy |
| --- | --- | --- |
| 0 | 49.4 | 57 |
| 8 | 66.7 | 61.4 |
| 20 | 69.6 | 53.4 |

11- To assist in the review process, add line numbering to the document.

Thank you for your comment, completions are made both page numbering and line numbering.

**Reviewer #2:** Summary
The study was conducted to examine the effects of MediYoga (MY), in comparison to Physiotherapy management techniques, on stress, anxiety and depression measurement scores among adults presenting with the associated symptoms such as musculoskeletal discomfort, headache and sleeping disorders. No clinical diagnoses were made on such conditions in this study; participants self-reported the symptoms using the self-administered questionnaires. Measures of stress constituted the primary outcome, whereas the anxiety and depression measures were secondary outcomes. On a 1:1 ratio, 46 participants were randomized either to MY (n=23) or PT (n=23). The MY was received once a week for 12 weeks, using a group therapy approach, each session lasting an hour. The PT intervention was sub-categorized further into 6; body and mind (=4), exercise therapy (4), combination of body and mind and exercise therapy (n=8), combination of exercise therapy and pain management (n=2), and ‘no treatment’ (n=5 [n=3 just assessment?]). The PT was delivered to each participant, individually, one to three times a week for ‘several weeks?’, each session lasting 30-45 minutes. Participants were assessed at baseline, 8- and 20-weeks. All treatments were delivered by Physiotherapists. Participants on medications or psychological related treatment continued with their therapies alongside the interventions in this study. Whereas those who were receiving or had received MY and PT interventions within the past 3-months were excluded from participation.
Authors report that at 8-weeks, nearly half of the participants were lost to follow-up. Authors claim that MY is more effective in improving stress, anxiety and HR-QOL than PT interventions. Authors report poor adherence to the intervention as the study’s major limiting factor.


Major issues
1. On page 6, it is not clear what criteria was used to allocate participants to the various conventional PT interventions**.**

Thank you for your comment. We have tried to clarify this in the method section. There was only one PT intervention, see page 6 line 139.

Physiotherapy treatment as usual and the description of four categories of physiotherapy are moved to Result section, see page 9 line 219. The four categories of variations in physiotherapy were analyzed and described after the study was closed clinically.

The rationale of having other participants not receiving any conventional PT intervention (within the PT as standard carearm) is not clear They didn’t show up to treatment sessions.
3. ‘Age was considered a confounder, but never met the inclusion criteria for confounders hence the decision to not adjust ?
the models for age’. Was this, in any way, due to the recruitment (by chance)???

Thank you for your comment se answer under 3a.

a. Did you consider matching the participants, in both arms, for age, gender and weight? (Potential confounders for stress, anxiety, and depression [severity/progressiveness/recovery])

In this study the inclusion criteria were self-reported symptoms from stress, and also stress-related symptoms like anxiety and musculoskeletal discomfort. We choose these inclusion criteria to stay close to what the patient flow looks like in primary care. We decided on an RCT design without any stratifications. The results showed that only two men participated in the study and both happened to be allocated to PT-group. However, fewer men seek primary care for stress and anxiety. We tested age to be a confounder, but it was not. Weight was not considered was not believed to be a decisive factor for how the outcome of compliance would be in the study regardless of group affiliation.

b) Could the inability to match the participants limit the interpretation/generalizability of the results**?**

In our judgement, using an RCT in which a block-randomized generated list with five in each block produced from a computer-generated list for the randomization, we rather think the high drop-out rate limits the interpretation/generalizability.

4. The comparison group (PT) was heterogeneous. Different participants received different PT conventional interventions
(also with varying dosage?), which might affect the findings differently as well (as reported in your results). This makes the conclusion that MY is more effective to 'PT' arguable due to lack of standardization of the comparison intervention. Yes it was as the comparison group was PT treatment as usual, ie, the treatment that would have been chosen for the patient regardless of he/she participated in the study. However, we were able to follow and report this treatment as usual and chose to categories received treatment. We have tried to clarify this also under question number 1 and in the text on , method section page 6 line 139 and result section, see page 9 line 219.

Comparing MY to each sub-group of PT interventions (bearing in mind the sample size calculation approach) would have been ideal. You may wish to incorporate this in the interpretation and discussion of the results. It might have been possible but was not the original research question and hence did not have the simple size to support this sub-grouping. We hope that the answers to question number 1 have clarified the sub-grouping.

Minor issues
1. Line 8 of page 2, consider removing the space between ‘b ut’ (for ‘but’) Thank you for noticing, completed see page 2 line 45.
2. Line 5 of page 5, consider replacing ‘control’ with usual/standard care Thank you for your comment, see page 5 line 117. We have tried to replace in the document to “physiotherapy treatment as usual” where it is justified.

3. Consider putting SI unit for age in table 1 on page 5, Changed in accordance to suggestion.

4. ‘Participation during 8-weeks in mean’ column in table 1, see table 1 is this for number of sessions? Yes, it is for number of sessions, completed in table 1, see page 5, line 123.

5. Line 5 of page 7 ‘of practical reasons’….. may benefit from a sentence reconstruction. The sentence is now reconstructed, see page 6, line number 149.

6. Line 2 on page 8 ‘for use with people with…’ may benefit from a sentence reconstruction.

The sentence has been reconstructed for clarity, page 6 line 163.

7. Line 4 on page 8 ‘to access’, did you want to say ‘to assess’? that’s right now completed see page 7line 165
8. Lines 8-9 on page 10; the sentence is not clear, may benefit from a sentence reconstruction.

The sentence has been reconstructed for clarity, page 13 line 272.

The major findings were that for MY as a group treatment intervention for 8 weeks, showed reduced levels of stress and anxiety and increased HRQoL compared to PT treatment as usual.”

9. Lines 8-10 on page 15 may benefit from a sentence reconstruction The sentence has been reconstructed for clarity, page 13 line 278.

As treatment in this category has similar impact in the autonomic nervous system as do the MY treatment, this may explain why the differences were not so great between the MY group and the PT group regarding the experience of stress and depression.

10. **Lines 18-19 on page 15 may benefit from a sentence reconstruction**
a. Do you mean there was no statistical difference between the two groups? Or no statistical changes within groups?

We have tried to clarify in the manuscript see page 13 line 281, There was no statistical difference between the MY group and the PT group.

b. The trend of improvement, was it for MY or PT or in both groups? The results (using mixed-effects linear models) show the differences between the groups, the trend of improvement was for MediYoga.

11. Lines 7-8 on page 16, sentence starting wit ‘consistent with previous research, as in randomized……16’, may benefit from a
sentence reconstruction

The sentence has been reconstructed for clarity, page 13 line 290. There was no statistically significant difference between the MY and PT groups regarding the outcome HADS depression. This result is consistent with previous research as in a randomized controlled trial where MY was allocated for one hour once a week over a 12-week period, in addition to the standard care.”

**Reviewer #3:** Summary of short comings
1. Page 1; Write something in the abstract on how data was analysed
How data was analysed “Mixed effect models was used for the analysis.” has been added to the abstract, see page 1 line 28.
SE = standard error depression (P = 0.08)”. This sentence is a little confusing. The results were not statistically significant, yet you indicate that there was a trend using that P value.

Yes, we interpreted the results for HADS depression, as you say not to be statistically significant, but that there was a trend for the interaction (p<0.10).

3. Page 5, line 3: The font color is not consistent with the rest of the text. Completed, see page 5 line 115.
4. Page 5, line 5: There is a hyphen as a sign of correction… need to remove it ? Completed see page 5 line 117.
5. Page 7: Explain how “no assessment and no treatment” is part of PT intervention group. No assessment and no treatment - after randomization three participants didn´t show up to their first assessment to the physiotherapist and they didn’t show up for treatment either.
6. Page 7 & 8: indicate the psychometric properties of the instruments used?

Thank you for your comment. We have tried to explain the psychometric properties of the instruments for the primary and secondary outcomes on page 6-8 line 153-196.

7. Page 9 paragraph 2: stick to third person instead of first person speech. We have tried to find this sentence but we don,t know where in the manuscript we need to change?

8. Page 10-14: Table 4 is too long, crowded and confusing. If possible find a way to separate the results for each outcome instead of combining all outcomes in one table. Of course there will be too many tables but if there is a provision of submitting tables separately, would be good.

Thank you for your comment The fact that the table is long is due to many outcomes, but only includes the basic information of each model for each of the outcomes.

9. Is there a reason for presenting the intercept in Table 4? I don’t see the results of intercept being explained in this manuscript.

According to our professor and statistician (second author) one should show the entire model in Table 4 and therefore the intercept should remain there. Even if you do not interpret it, it is included in the model.

10. There is some inconsistence in the reference list. For instance for reference 25, there is a need to add other authors. Thank you for your comment reference 25 is now completed and also other inconsistencies in the reference list.
11. Table 4: it should be “95%CI” and not just “CI” Thank you for your comment, completions are made in Table 5 for 95%CI page 12 se line number 258.

12. Table 4: Mean should be written together with standard deviation.

Now a foot note is added to Table 4, page 12 line 256 explaining that SE = standard error

13. Page 15, line 4/5: The sentence doesn’t seem complete. HADS had significant difference in comparison to which variable? Completed page 12 line 266.

For HADS depression there was a significant difference (*P* = 0.03) between the group of those who answered the questionnaire at 8 weeks (n = 20) and those who had dropped out at 8 weeks (n = 17).
14. Page 17, line 2: Majority of the interventions require participants to travel to the clinical site. However, was there something very specific to your study that contributed to the high dropouts?

There was not a long distance to the rehabilitation clinic and hence probably not the reason for the dropout in the current study. According to our experience the drop-out could rather be derived to the psychological state of each individual. Many of the patients expressed that it was very much the day-to-day form that determined whether they would be able to go out for treatment or follow-up appointments. The majority of the patients in the study described that some days they had difficulty mobilizing strength to get outside the home.
