# Supplementary material for: MediYoga compared to physiotherapy treatment as usual for patients with stress-related symptoms in primary care rehabilitation: A randomized controlled trial
Source: PLoS One. 2024 Jun 13;19(6):e0300756. doi: 10.1371/journal.pone.0300756 (PMC11175516; doi:10.1371/journal.pone.0300756)
Supplement: Attachment — Submitted filename: 240127 Response to reviewers.docx [file pone.0300756.s010.docx]

**Response to reviewers**:

**Review Comments to the Author**

Please use the space provided to explain your answers to the questions above. You may

also include additional comments for the author, including concerns about dual

publication, research ethics, or publication ethics. (Please upload your review as an

attachment if it exceeds 20,000 characters)

Reviewer #1: Minor revisions:
1- Line 212: Variance component is not a covariance structure. The three most common underlying covariance structures used in mixed effects linear models are: unstructured, autoregressive, and compound symmetry. Thank you for your comment, see page 8, line number 208-213 where this has been clarified. The covariance structure used in the mixed effects model is the *compound symmetry* for the random effects.

2- Pertaining to the statistical power calculation, within the manuscript indicate that the t-test was the statistical method which attained 80% power. Prior Comment: "Statistical Power Calculation: State the statistical testing method which attains 80% power. Perhaps it is a t-test." And the response: "Yes, the statistical power calculation was done with a t-test. Thank you for your comment, see page 8, line 198. A t-test was used when power was calculated for the primary outcome general stress level, measured with the PSS.

We also noticed an error which has been corrected. On page 4 line 109 it says “A block-randomized generated list with five in each block, produced from a computer-generated list, was used for the randomization.” It should be “A block-randomization generated list with four blocks with ten in each block, produced…” This has been corrected in manuscript.

Reference list

We have also reviewed our reference list to ensure that it is complete and correct. We have made changes in page numbers in ref no 2, 7, 9, 19, 21 och 27.
